# Novel Factors in the Pathogenesis of Psoriasis and Potential Drug Candidates Are Found with Systems Biology Approach

**DOI:** 10.1371/journal.pone.0080751

**Published:** 2013-11-26

**Authors:** Máté Manczinger, Lajos Kemény

**Affiliations:** 1 Department of Dermatology and Allergology, University of Szeged, Szeged, Hungary; 2 Dermatological Research Group of the Hungarian Academy of Sciences, Szeged, Hungary; Semmelweis University, Hungary

## Abstract

Psoriasis is a multifactorial inflammatory skin disease characterized by increased proliferation of keratinocytes, activation of immune cells and susceptibility to metabolic syndrome. Systems biology approach makes it possible to reveal novel important factors in the pathogenesis of the disease. Protein-protein, protein-DNA, merged (containing both protein-protein and protein-DNA interactions) and chemical-protein interaction networks were constructed consisting of differentially expressed genes (DEG) between lesional and non-lesional skin samples of psoriatic patients and/or the encoded proteins. DEGs were determined by microarray meta-analysis using MetaOMICS package. We used STRING for protein-protein, CisRED for protein-DNA and STITCH for chemical-protein interaction network construction. General network-, cluster- and motif-analysis were carried out in each network. Many DEG-coded proteins (CCNA2, FYN, PIK3R1, CTGF, F3) and transcription factors (AR, TFDP1, MEF2A, MECOM) were identified as central nodes, suggesting their potential role in psoriasis pathogenesis. CCNA2, TFDP1 and MECOM might play role in the hyperproliferation of keratinocytes, whereas FYN may be involved in the disturbed immunity in psoriasis. AR can be an important link between inflammation and insulin resistance, while MEF2A has role in insulin signaling. A controller sub-network was constructed from interlinked positive feedback loops that with the capability to maintain psoriatic lesional phenotype. Analysis of chemical-protein interaction networks detected 34 drugs with previously confirmed disease-modifying effects, 23 drugs with some experimental evidences, and 21 drugs with case reports suggesting their positive or negative effects. In addition, 99 unpublished drug candidates were also found, that might serve future treatments for psoriasis.

## Introduction

Psoriasis is a multifactorial inflammatory skin disease. A recent systematic review reported a prevalence from 0% (Taiwan) to 2.1% (Italy) in children and from 0.91% (United States) to 8.5% (Norway) in adults.[1] Genetic predisposition and environmental factors are both important in disease etiology. Several genome-wide association studies have been carried out and until now 36 susceptibility loci have been identified.[2] Environmental triggers are also reported such as drugs, smoking, mental stress, skin injury, Streptococcal infection, hormonal changes etc.[3] Psoriasis is an immune-mediated disease. Important immune cells and cytokines have been identified in disease pathogenesis such as IL6, IL17A, TNF etc.[4] Autoimmune basis for chronic inflammation is supposed, although no consistent antigen has been found. Patients with psoriasis have higher risk for metabolic syndrome, and risk increases with disease severity. Both diseases have immunological basis with common cytokines and genetic risk loci like CDKAL1.[5] Keratinocyte hyperproliferation is present in lesional phenotype and is responsible for scale formation. Keratinocyte differentiation markers like keratin 1 and keratin 10 are downregulated and parakeratosis (keratinocytes with nuclei in the stratum granulosum) is also present.[3]

Psoriasis is one of the most studied skin diseases. By now more than 34000 hits are available in PubMed for the keyword “psoriasis” and the number is increasing. No spontaneous psoriasis-like skin disease is known in animals. Induced mouse models are available which are similar, but not the same as psoriasis in human.[6] Therefore drug discovery is difficult in such models what makes in silico analysis more essential. “Omics” data gives the opportunity to examine the disease with systems biology approach.

Stationary changes in gene expression are responsible for fixing phenotypes such as lesional skin areas in psoriasis. Several microarray studies have been carried out to characterize gene expression in healthy and psoriatic skin samples (Table 1). Microarray meta-analysis gives the opportunity to evade biological, regional, and study design-caused variation between studies.[7] Network analysis is a novel and highly developing area of systems biology. Considering gene expression data it is possible to explain alterations in intracellular processes with the analysis of protein-protein and protein-DNA (or gene regulatory) interaction networks. These networks consist of proteins and/or regulated genes as nodes and undirected or directed edges between them. Network centralities like degree or stress are suitable for ranking nodes. Total edge number belong to one node equals its degree in undirected networks. Nodes have in- and out-degrees based on edge directions in directed networks. Degree distribution follows a scale-free power law distribution in biological networks. This fact indicates that highly connected vertices have a large chance of occurring. Nodes with highest degree are called hubs and are essential in network stability.[8] Stress centrality indicates the number of shortest paths (from all shortest paths between any two nodes in the network) passing through the given node thus the capability of a protein for holding together communicating nodes.[9] Interconnecting nodes make up network motifs. Several, such as feed-forward or bifan motif are significantly enriched in biological networks compared to random networks. These elements have important role in network dynamics.[10]

**Table 1:** Study information and QC measure summary.

	Study	MIAME	GEO ID	Platform/Chip	NL	L	IQC	EQC	CQCg	CQCp	AQCg	AQCp	Rank
1	Gudjonsson et al.[110]	Available	GSE13355	GPL570/Affymetrix HU133 Plus 2.0	54	53	4.18	4	307.65	307.65	95.2	292.19	2.17
2	Yao et al.[111]	Available	GSE14905	GPL570/Affymetrix HU133 Plus 2.0	27	32	5.58	4	307.65	307.65	81.32	185.34	2.67
3	Zaba et al.[112]	Available	GSE11903	GPL571/Affymetrix HU133A 2.0	15	12	7.34	3	307.65	307.65	79.24	260.95	2.75
4	Suarez-Farinas et al.[113]	Available	GSE30999	GPL570/Affymetrix HU133 Plus 2.0	79	80	0.86*	4	307.65	307.65	33	193.93	3.67
5	Reischl et al.[114]	Available	GSE6710	GPL96/Affymetrix HU133A	12	12	2.7	4	307.65	271.23	40.3	118.68	3.92
6	Johnson-Huang et al.[115]	Available	GSE30768	GPL571/Affymetrix HU133A 2.0	1	4	Excluded by Array Quality Metrics package						

MIAME information was available for all study Studies were downloaded from Gene Expression Omnibus (http://www.ncbi.nlm.nih.gov/geo/). All studies were carried out on Affymetrix platforms. Lesional and Non-Lesional sample count is shown. Stars in table indicate non-statistical significance of QC measures. Study no 6 was already excluded by sample filtering by arrayQualityMetrics. Other studies had high quality and no outlier study was present. IQC: Internal Quality index, EQC: External Quality index, CQCg and CQCp: Consistency Quality Control indexes, AQCg and AQCp: Accuracy Quality Control indexes, NL: non-lesional sample count, L: lesional sample count.

We hypothesized that it could be possible to find novel elements of psoriasis pathogenesis with detailed analysis of precisely constructed networks. Network motif enrichment caused by changes in gene expression could have important role in disease development and sustainment. It could be also possible to detect potential drug candidates by analyzing chemical–protein networks. Thus our goal was to construct reliable but yet detailed protein-protein, protein-DNA, merged (containing both protein-protein and protein-DNA interactions) and chemical-protein interaction networks consisting of differentially expressed genes (DEG) between lesional and non-lesional skin samples and/or the coded proteins. Detailed analysis of these networks could help us to reveal novel players in disease pathomechanism and to identify network motifs and sub-networks with the ability to sustain lesional phenotype.

## Methods

### Microarray Meta-analysis

Six microarray studies examining lesional and non-lesional skin biopsy samples of psoriatic patients were found in Gene Expression Omnibus (GEO) (Table 1). “Minimum Information About a Microarray Experiment” (MIAME) was available for each study. Only non-lesional and lesional samples from affected individuals were used for analysis, samples from healthy people were excluded. Raw.CEL files were downloaded and quality of each sample was assessed with the R package arrayQualityMetrics.[11] This package defines sample quality with 5 different methods and generates plots for outlier detection. A sample was excluded if it was obviously an outlier in at least 1 measure or had borderline values in at least 2 measures (analysis results are in Dataset S1 compressed file; outliers and argument of exclusion is listed in Table S1). Raw data normalization of remaining samples was carried out with the R package Easy Microarray data Analysis (EMA).[12] GCRMA normalization method was used and probe sets with expression level below 3.5 were discarded. Probe set with the highest interquartile range (IQR) was chosen for common HUGO Gene Nomenclature Committee (HGNC) gene identifiers. Original findings were confirmed with published statistics. For this EMA was used after GCRMA normalization. More DEGs were found in some cases, which might be caused by the pre-filtering process with arrayQualityMetrics (Table S2). The R package MetaQC was used for filtering out low quality studies.[13] The fifty most prevalent gene set were chosen with the software Gene Set Enrichment Analysis (GSEA) and used for external quality control (EQC) score calculation.[14] GSEA was carried out for each study with the following settings: 1000 permutations; minimum set size was 5 and the gene set database was c2.all.4.0.symbols. The resultant study-level p values of a gene set were combined with Fisher's combined probability test. The fifty gene sets with the lowest meta-analysis p value were chosen as input for EQC score calculation. C2.all.4.0.symbols gene set database was chosen as input for consistency quality control (CQCp) value calculation. GSEA input expression matrices contained gene IDs that were present in all studies after EMA filtering. MetaDE package was used to determine DEGs in lesional samples compared to non-lesional ones.[15] DEG p value in individual studies was calculated by two sample T test with unequal variances. Fisher's combined probability test was chosen for meta-analysis statistical method.[16] Fold change of gene expression was given by the ratio between geometrical means of gene expression in lesional and non-lesional samples.[17] Genes with false discovery rate (FDR) less than 0.001 and with fold change higher than 1.5 or less than −1.5 were accepted as DEGs.

### Construction of protein-protein, protein-DNA and chemical-protein interaction networks

STRING database 9.0 was used as resource for protein-protein interactions (PPI).[18] Both directed and undirected networks were created by selecting all interactions between DEG – coded proteins in downloaded raw data. Interaction confidence score cutoff was 900 (“highest confidence” group) in case of undirected and 800 (containing a part of “high confidence” and all “highest confidence” interactions) in case of directed interactions. Only directed interactions with “activation” or “ptmod” actions were used. Chemical-protein interactions between potential drugs, intra- and extracellular compounds and DEG-coded proteins were collected from STITCH database 3.1.[19] The way of interaction confidence score calculation is the same in this database as in STRING thus interactions with the described confidence score cutoff values were selected for network construction. Protein-DNA interaction (PDI) network consisting of DEGs and DEG-coded transcription factors (TF) was created using cis-Regulatory Element Database (CisRED).[20] Regulatory element motifs with 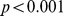 were collected from DEG promoter regions. Motifs were coupled with TFs or TF complexes using TRANSFAC and JASPAR databases.[21], [22] Motifs without respective TFs were excluded. Merged DEG-derived network containing PPI and PDI interactions and a network containing only DEG-coded TFs were also generated. Complete PPI, PDI, merged, TF-TF and chemical-protein interaction networks were created for controls using all available interactions in databases with the same statistical threshold as in DEG-derived network construction.

### General network analysis, identification of central nodes and motif detection

General network analysis and node centrality value calculation were carried out with NetworkAnalyzer Cytoscape plugin.[23] Isolated nodes and node groups (without connection with the main PPI network) were deleted from graph in order to evade false results. Curve fitting on node degree and stress value distributions was done with MATLAB Curve Fitting Tool (MATLAB R2012b, The Mathworks Inc., Natick, MA). Curve of power law distribution was assessed with Trust-Region algorithm. Goodness of fitting was assessed by R-square and corrected R-square values which prove power law distribution of these node centralities (Table 2). As power law distribution is asymmetric with a long tail, nodes with centralities above average cannot be assessed using arithmetic mean. A variable with a power-law distribution has a probability 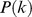 of taking a value 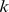 following the function 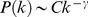, where 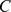 is constant. First moment (mean value) of a power-law distributed quantity equals:
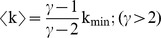



**Table 2:** Results of node centrality analysis.

Network	Centrality	Curve	Cutoff	R-square	Adjusted R-square
PPI Undirected	*Degree*	0.8555◯-1.649	27.21	0.9957	0.9956
	*Stress*	47.1◯-0.8034	427072.25	0.9795	0.9793
PPI Directed	*In-Degree*	0.5925◯-1.808	5.100152	0.9969	0.9968
	*Out-Degree*	0.5462◯-1.759	23.493461	0.9983	0.9983
	*Stress*	15.34◯-0.961	8504.103	0.8753	0.8748
PDI	*Out-Degree*	13280◯-1.367	287.20865	0.9252	0.9002
CPI Undirected	*Degree*	0.8314◯-3.168	14.761	1	1
	*Stress*	2.41e1 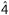 -2.432	6.63	0.9811	0.9811
CPI Directed	*Out-Degree*	0.7859◯-2.132	8.5757576	1	1

Distribution of node centrality values were assessed by curve fitting. Curve equations, goodness of fit (R-square and adjusted R-square) and the resultant cutoff values are shown. CPI: chemical – protein interaction network.

Second moment (variance) of a power-law distributed quantity equals:
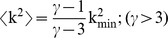



The sum of first and second moment (mean value and variance) was used as cutoff for centralities with distribution exponent 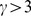. Expression of variance becomes infinite, when 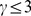, thus only first moment (mean value) was used as cutoff for centralities with distribution exponent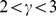. [24] Expression of mean value becomes infinite, if 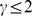. In this case weighted mean was used to assess cutoff with the following formula:
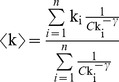



As bidirectional connections are available in undirected PPI network, stress centrality is independent from edge directions thus both degree and stress had to be above cutoff for central protein selection. As directed networks contain unidirectional interactions, low stress values (i. e. low number of shortest paths cross through the node) can be caused by the dominance of incoming (in-degree) or outgoing (out-degree) interactions. Important nodes with high in-degree or out-degree can still have low stress centrality thus either out-degree or in-degree or stress had to be above cutoff in directed PPI network. As TFs have mainly outgoing interactions, out-degree was used for TF prioritization. Similarly to PPI networks degree and stress had to be above cutoff in undirected chemical - protein interaction network. Drugs with more targets in DEG-derived PPI-networks may have bigger disease modifying effect thus out-degree had to be above cutoff in directed chemical – protein interaction network for drug prioritization (Table 2).

NetMODE software was used for network motif statistical analysis. Frequency of 3 or 4 node motifs in DEG-derived and complete control networks were compared with 1000 random graphs. *Local constant switching mode* was used for edge switching method during random network generation. NetMODE p value indicates the number of random networks in which a motif occurred more often than in the input network, divided by total number of random networks. 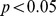 was used as cutoff.[25] Respective sub-networks of enriched motifs were identified with NetMatch Cytoscape plugin.[26] jActiveModules and ClusterONE were used for network module and protein complex detection. ClusterONE analysis was carried out with *minimum cluster size* of 3 with unweighted edges and default advanced parameters. jActiveModules considers gene expression for module search. Input gene expression values have to be between 0 and 1 so normalized expression values got with EMA were scaled between these numbers.[27], [28] Functional description of node groups was done with BinGO (“Biological function” GO terms were selected, FDR<0.001 was used for term enrichment).[29]

## Results

### Detection of DEGs with microarray meta-analysis

**Figure 1:**
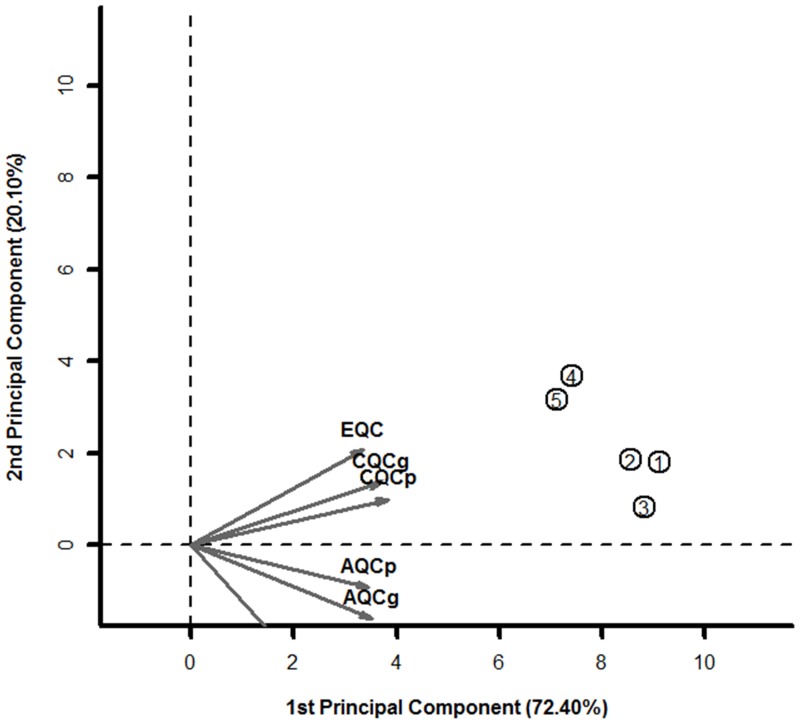
PCA biplot numbers on PCA biplot represents studies in Table 1. Study number placed opposite to quality measure axes are of low quality and should be excluded. No outlier study was detected.

In order to get reliable data about gene expression in lesional psoriatic skin samples microarray meta-analysis was carried out. The study by Johnson-Huang et al. was already excluded after sample quality analysis with arrayQualityMetrics package, because at least two samples from one phenotype group are needed for MetaQC analysis and only one non-lesional sample remained after sample filtering. The overall quality of each study was assessed by MetaQC.[13] The software calculated six quality control (QC) measures then created principal component analysis (PCA) biplot and standardized mean rank summary (SMR) score to help in the identification of problematic studies. It was described by authors, that if a study is on the opposite side of arrows in the PCA biplot and has large SMR scores, it's strongly suggested to be excluded from meta-analysis. In contrary, if a study is on the same side of arrows in the PCA biplot and has small SMR scores, it should be included. All five studies were defined as usable based on quality values (Table 1, Figure 1). DEGs were identified by MetaDE.[15] 2307 upregulated and 3056 downregulated genes were found in lesional skin samples compared to non-lesional ones (Table S3). The relatively high number of DEGs can be the result of filtering out low quality samples, which could increase variance and using lower fold change cutoff values than in original studies. DEGs were used for network construction.

### General Network analysis

**Figure 2:**
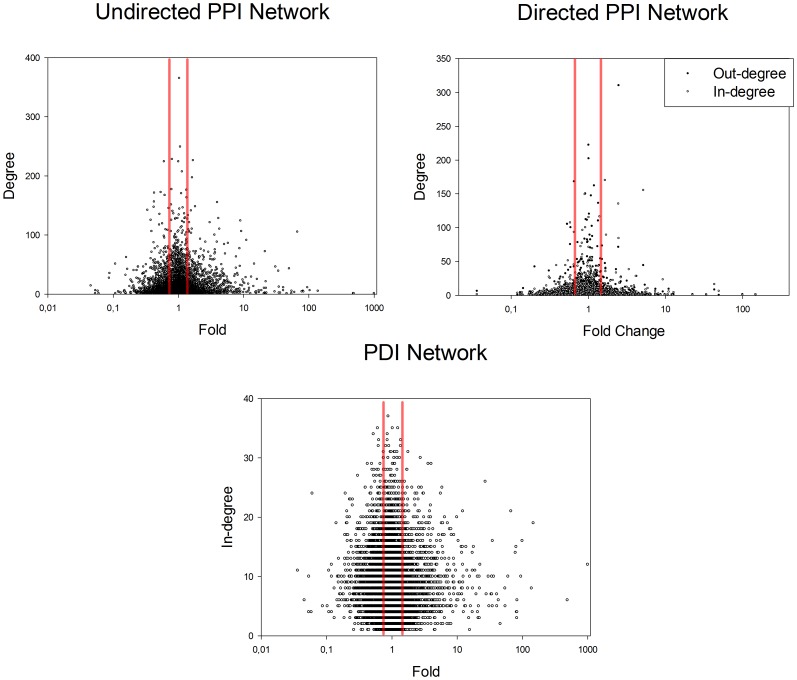
Degree-Fold Change relationship. Nodes with higher degree has lower fold change of gene expression in all network types. Genes between red lines have higher average degree and are filtered out from network analysis. Remaining nodes in DEG-derived networks have lower average degree and connectivity.

Undirected and directed PPI networks with DEG – coded proteins, directed PDI networks with DEG – coded TFs and regulated DEGs and merged directed networks containing both PPIs and PDIs were created. A TF-TF network consisting of DEG-coded TFs was also generated. The Cytoscape plugin NetworkAnalyzer calculated main network properties for both DEG-derived and control complete networks (Table 3). DEG – derived networks had higher diameter (i. e. the length of the longest shortest path in the network) and average shortest path length than control full networks. This may be caused by the inverse correlation of node degree and fold change.[30] Nodes with lower fold change has higher degree. Genes with fold change under cutoff are filtered out from DEG derived networks (between red lines on Figure 2). The remaining nodes has smaller average degree, therefore connectivity of the network is lower resulting in higher diameter and average shortest path length value.

**Table 3:** Results of general network analysis.

Network	Nodes	Edges	Diameter	Average shortest path
PPI Undirected	1614 *(9412)*	5156 *(55039)*	14 *(12)*	4.79 *(4.45)*
PPI Directed	464 *(4040)*	815 *(13377)*	14 *(12)*	5.26 *(4.35)*
PDI	2840 *(15839)*	6398 *(123210)*	10 *(7)*	3.69 *(3.029)*

DEG derived and control networks has similar attributes, but average shortest path length and network diameter is lower in DEG derived networks, which can be explained by lower connectivity (Figure 2). Values for control networks are in brackets.

### Determination of hubs in DEG-derived networks

Most important nodes of DEG-derived networks were determined using degree and/or stress centralities (Table 2, full list of nodes and centralities is in Table S4). Numerous already published psoriasis-associated protein-coding genes were found (Table 4). CCNA2, FYN and PIK3R1 proteins are present in top rated hubs in undirected PPI network and are yet unpublished in association with the disease. CCNA2 have role in mitosis regulation.[31] FYN is important in interferon gamma (IFN gamma) signaling, while PIK3R1 is important in insulin-stimulated glucose uptake.[32], [33] FYN could be found in jActiveModules cluster with the 2^nd^ highest score while PIK3R1 were found in cluster with the 3^rd^ highest score (Figure S1, S2). Taking account BinGO results these clusters are responsible for signaling and for immune regulation as well (Table S5). A highly connected chemokine-chemokine receptor cluster was also found with ClusterONE analysis (Figure S3). Central nodes in directed and undirected PPI networks showed overlap (Table 4). CTGF is in top ranked proteins and yet not associated with psoriasis. CTGF is responsible for fibrosis downstream of TGFβ signaling. Downregulation of CTGF by psoriasis-associated cytokines INFγ and TNFα is already published.[34]

**Table 4:** Top rated nodes in DEG-derived networks.

PPI Undirected		PPI Directed		PDI	
Name	Fold change	Name	Fold change	Name	Fold change
IL8	67.31113193	IL8	67.31113193	**TFDP1**	4.612130627
CCNB1	11.13277565	BIRC5	9.309154577	**MECOM**	1.705869235
BIRC5	9.309154577	MMP1	7.446458555	**AR**	−1.649992095
STAT1	9.038900879	SOD2	7.198087989	NF1	−1.707954442
**CCNA2**	8.737535122	IL1B	4.293906976	**MEF2A**	−1.738635445
CXCR4	5.109553129	STAT3	3.965626652		
IL1B	4.293906976	MMP9	3.661047085		
MAPK14	4.152927326	SOCS3	3.315643007		
STAT3	3.965626652	HMOX1	3.207443671		
MMP9	3.661047085	CCL2	2.896844503		
LCK	3.609090653	BAX	1.9009731		
AURKB	2.493884913	ICAM1	1.722246429		
MAPK1	1.820524831	CD69	1.721780507		
MYC	1.690987073	MYC	1.690987073		
NFKB1	1.636019496	CD86	1.676295675		
PCNA	1.623673041	CD28	1.640633244		
CDKN1A	1.583889601	NFKB1	1.636019496		
HDAC1	1.57828429	EGFR	−1.607280925		
CYP1A1	−1.595883159	CTNNB1	−1.648110677		
EGFR	−1.607280925	FN1	−1.75413351		
CREBBP	−1.626480892	EDN1	−1.836157927		
CTNNB1	−1.648110677	SP1	−1.923552267		
FN1	−1.75413351	**CTGF**	−2.037178621		
**FYN**	−1.849385591	NFATC1	−2.187942784		
SP1	−1.923552267	IRS1	−2.277490062		
SMAD4	−1.95145712	INS−IGF2	−2.33005624		
INS-IGF2	−2.33005624	CCND1	−2.341844947		
CCND1	−2.341844947	FOS	−2.362430819		
FOS	−2.362430819	PPARG	−2.556455049		
PPARG	−2.556455049	BCL2	−2.632996792		
BCL2	−2.632996792	**F3**	−3.835078706		
**PIK3R1**	−2.955639724	LEP	−6.266827433		

Central proteins with centrality value(s) above cutoff are listed. Fold change between gene expression in lesional and non-lesional samples are also shown. Proteins with bold characters are yet non-published in terms of psoriasis.

PDI network contained DEG-coded TFs and regulated DEGs as nodes and directed edges pointing from the TFs to the regulated genes. TFs were ranked using out-degree centrality. Androgen receptor (AR) and TFDP1 were the highest ranked nodes. AR is a TF, regulating genes that have immunological functions and role in carbohydrate metabolism.[35], [36] TFDP1 controls cell cycle progression and is yet not associated with psoriasis.[37] BinGO analysis of TFDP1-regulated genes prove its central role in cell cycle activation (Table S5). MECOM and MEF2A are TFs above centrality cutoff and yet not associated with psoriasis. MECOM have role in cell proliferation and is associated with chronic myeloid leukemia.[38] MEF2A is responsible for the insulin dependent glucose transporter GLUT4 expression and is downregulated in insulin deficient diabetes mellitus.[39]

### Motif analysis in DEG-derived networks

**Figure 3:**
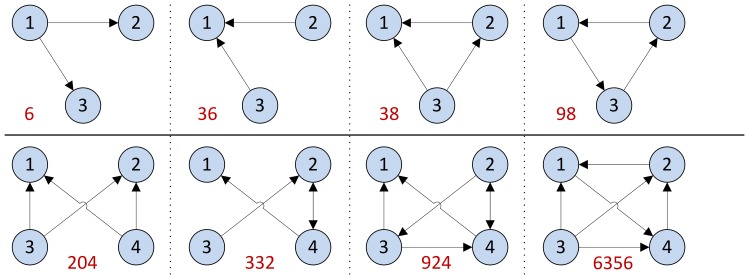
Network motifs with 3 or 4 nodes. Analysis results of the respective motif can be found in Table 5.

Motifs consisting of 3 or 4 nodes were analyzed in directed DEG-derived and control networks as well (Table 5, Figure 3). Analysis found motifs which were enriched in directed DEG-derived but were absent in control networks or vice versa. Some were already generally described in biological systems like convergent (no. 36), divergent (no. 6) and bifan (no. 204) motifs, but yet non-examined ones were detected like motif no. 924 in directed PPI networks, no. 332 in TF-TF networks and no. 6356 in merged networks etc. Cause of missing convergent, divergent and bifan motifs in DEG derived directed PPI or PDI networks compared to control was not investigated as uncertainty is present about the role of these network motifs in biological systems.[10] Identifying nodes making up motif no. 924 resulted in the high occurrence of central proteins found before. These proteins were associated with the immune system and carbohydrate metabolism. Motif 332 is enriched in the TF network of lesional skin. This motif is based on the TFDP1–AR reciprocal regulation. Importance of these TFs is already mentioned.

**Table 5:** Summary of network motif analysis.

	PPI directed		PDI		PDI +PPI	
Motif no.	Psoriasis	Full	Psoriasis	Full	Psoriasis	Full
6 (divergent)	0.705	0.031	0.168	0.974	0.908	0.952
36 (convergent)	0.997	0.972	0.826	0.023	0.083	0.045
38 (feed-forward)	0	0	0.073	0.978	0.941	0.998
98 (feedback)	0.329	0.242	0.518	0.233	0.064	0.046
204 (bifan)	0.255	0	0.483	0.082	0.872	0.041
332	0.958	0.162	0.042 (TF network)	0.838 (TF network)	0.41	0.067
924	0.007	0.292	N/A	0.305	0.794	0.17
6356	0.025	0.02	N/A	0.916	0.001	0.512

Numbers are p values of motif enrichment compared to 1000 random networks. Values with bold characters are below 0.05 and thus significant. Significant enrichment was only found in TF-TF networks in case of motif no. 332. Network motif pictures are in Figure 3.

An interesting result of motif analysis is the enrichment of feedback loops containing 3 nodes in merged networks compared to separate ones and the enrichment of motif no. 6356 in DEG-derived merged network compared to control. Motif no. 6356 consist of a positive feedback loop and all nodes of the loop are controlled by another separated node like IL1B or AR.

### Controller sub-network construction

Both lesional and non-lesional skin areas can be found on patients at the same time. We wanted to highlight nodes which may be important in the “all or none” switch in lesional skin areas and sustain this phenotype for a long time. It has been argued that hubs in intracellular regulatory networks are enriched with either positive or negative regulatory links and cause much more positive feedback loops than negative ones.[40] It is also proven that positive feedback loops have fundamental role in maintaining autoimmune and autoinflammatory disease states.[41] Enrichment of motif no. 6356 consisting of a positive feedback loop with all nodes controlled by a separated one also suggests central role of positive feedback loops in lesional skin which may be activated by important central proteins like AR or IL1B. This is published that in biological systems interlinked slow and fast positive feedback loops allow systems to convert graded inputs (like several environmental and genetic factors in a psoriatic individual) into decisive all or none outputs (like lesional skin phenotype).[42] Transcriptional regulation needs time so we hypothesized that slow positive feedback loops may consist of at least one gene regulatory interaction. Fast loops may consist of only PPIs. Transcriptional changes of nodes in these loops may be able to sustain the “switched on” state.

**Figure 4:**
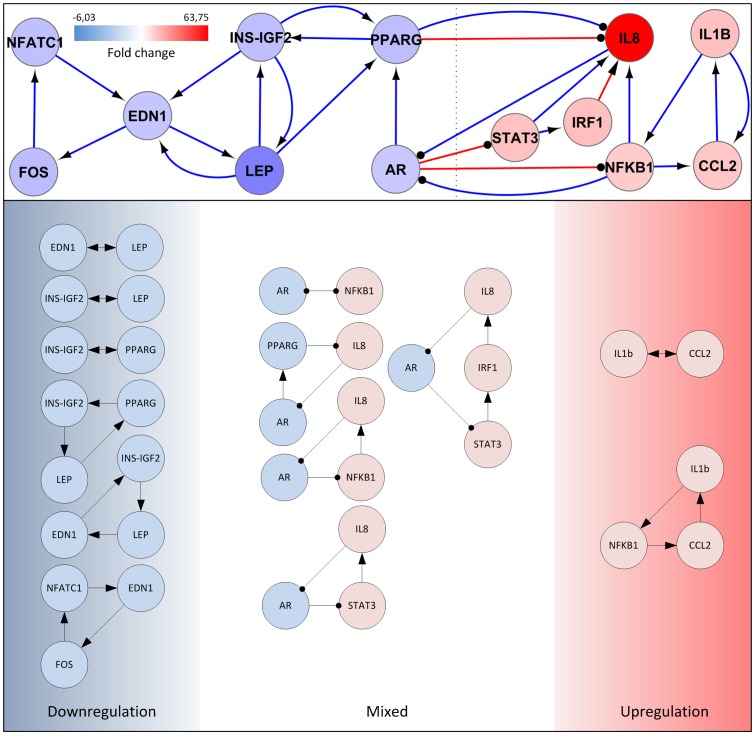
Positive feedback loops and the merged controller sub-network in lesional psoriatic skin. Individual positive feedback loops with 2, 3 or 4 nodes are shown. Node color is blue if the gene expression is decreased and red if increased. Merged controller sub-network is shown on the top. Node color is proportional with fold change. red line: gene regulatory interaction; blue line: protein-protein interaction; arrow-headed line: activation; bar-headed line: inhibition

In order to find most important slow and fast feedback loops containing 2, 3 or 4 nodes, a merged PPI and PDI network was constructed from proteins with centralities above cutoff value. All feedback loops were identified with NetMatch. A positive feedback loop was selected if and only if expression of all nodes changes in the direction of sustaining or suppressing the activity of the loop and “activation” or “inhibition” properties of all edges were proven by publications. Expression of all nodes was downregulated in two loops needed for carbohydrate metabolism: the INS-IGF2-EDN1-LEP-INS-IGF2 and the LEP-PPARG-INS-IGF2-LEP loop. The IL1B-NFKB1-CCL2-IL1B loop contained only upregulated nodes and has role in inflammation (Figure 4). The remaining loops contained inflammation and metabolism-related nodes as well. These may be key components in the metabolic-inflammatory interplay in the pathomechanism of psoriasis. “Slow” positive feedback loops containing gene regulatory interactions and “fast” loops containing only PPIs were also found. All positive feedback loops had common nodes, thus a merged network was generated containing interlinked slow and fast positive feedback loops (Figure 4). Transcriptional changes of all nodes and influence of all edges supported the sustainment of lesional phenotype in this sub-network. Boolean analysis of the resultant controller network was also performed. Nodes with downregulated expression got value of 0 and nodes with upregulated expression got value of 1. Future state of nodes was set based on interactions (Table 6). The output boolean values were the same as the input state values which prove the role of the controller network in the sustainment of present (lesional) phenotype. Chemical - protein interaction analysis further prove the importance of controller network.

**Table 6:** Boolean analysis of controller network.

	Input state	Relation	Future state(*)
NFATC* = FOS	0	0	0
FOS* = EDN1	0	0	0
EDN1* = NFATC1 and INS-IGF2 and LEP	0	0 and 0 and 0	0
INS-IGF2* = PPARG and LEP	0	0 and 0	0
LEP* = EDN1 and INS-IGF2	0	0 and 0	0
PPARG* = INS-IGF2 and LEP and AR	0	0 and 0 and 0	0
AR* = not (IL8 and NFKB1)	0	not (1 and 1)	0
STAT3* = not AR	1	not 0	1
IRF1* = STAT3	1	1	1
IL8* = not PPARG; STAT3 and IRF1 and NFKB1	1	not 0; 1 and 1 and 1	1
IL1B* = CCL2	1	1	1
NFKB1* = not AR; IL1B	1	not 0; 1	1
CCL2* = NFKB1 and IL1B	1	1 and 1	1

Logical relations can be seen in the first and third column. Input and future state of network is stationary.

### Analysis of chemical-protein interaction networks

Undirected and directed chemical-protein interaction networks were constructed using STITCH database, which contains interactions between proteins and chemical compounds (internal non-protein substances, drugs and environmental substances).[19] Drugs or potential drugs were filtered out from chemicals and ranked by degree and stress centrality in case of undirected and out degree centrality in case of directed networks (Table S4).

Top ranked drugs were grouped into Anatomical Therapeutic Chemical (ATC) classes (Table 7).[43] KEGG DRUG was used for classification.[44] Results show a big overlap between undirected and directed network analysis. Best rated drugs consisted of retinoic acid, cholecalciferol, costicosteroids, methotrexate, sirolimus and tacrolimus, which can be already found in psoriasis guidelines and large clinical trials have proved their effectiveness.[45]

**Table 7:** Published Drugs.

ATC Class	Drugs
STUDIES AVAILABLE	
*Retinoids for topical use in acne*	retinoic acid
*Corticosteroids*	dexamethasone, hydrocortisone, corticosterone, prednisolone
*H2 receptor antagonists*	cimetidine
*Immunosupressants*	sirolimus, tacrolimus
*Antiinflammatory and antirheumatic drugs*	indomethacin
*Blood glucose lowering drugs excl. insulines*	metformin, troglitazone, rosiglitazone, pioglitazone
*Intestinal anti-inflammatory agents*	sulfasalazine
*Vitamins*	cholecalciferol, folic acid
*Antimycobacterials*	rifampicin
*Mineral supplements*	selenium
*Antifungals for topical use*	salicylic acid
*Antineoplastic agents*	5-fluorouracil, methotrexate, paclitaxel, cycloheximide
*Cardiac stimulants excl. cardiac glycosides*	epinephrine-bitartrate, norepinephrine
*Lipid-modifying agents, plain*	simvastatin, atorvastatin-calcium
*Calcium channel blockers*	nifedipine
*Psychoanaleptics*	caffeine
*Thyroid therapy*	Liothyronine
*Drugs for obstructive airway diseases*	theophylline
*N/A*	berberine, curcumin, triptolide
EXPERIMENTAL EVIDENCE	
*Topical products for joint and muscular pain*	capsaicin
*Respiratory system*	N-acetyl-L-cysteine
*Antineoplastic agents*	Velcade, celecoxib
*Hormone antagonists and related agents*	tamoxifen
*Cardiac stimulants excl. cardiac glycosides*	isoproterenol
*Liver therapy*	glycyrrhizinic acid
*Antiinfectives and antiseptics, excl. combinations with corticosteroids*	arsenic
*Beta blocking agents*	propranolol
*Lipid-modifying agents, plain*	clofibrate, bezafibrate, fluvastatin, pravastatin
*Blood glucose lowering drugs excl. insulines*	ciglitazone
*N/A*	N-ethylmaleimide, baicalein, apigenin, SB 202190, monensin, rolipram, eflornithine, calphostin C, trichostatin A, rottlerin
CASE REPORTS	
*Antivirals for systemic use*	ritonavir
*Antiinflammatory and antirheumatic drugs*	diclofenac, ibuprofen, aspirin
*Antigout preparations*	colchicine
*Antiprotozoals*	chloroquine
*Ophtalmologicals*	atropine
*Antineoplastic agents*	cytarabine-hydrochloride, doxorubicin, cysplatin, imatinib, docetaxel, gefitinib
*Cardiac stimulants excl. cardiac glycosides*	phenylephrine
*Antiadrenergic agents, centrally acting*	clonidine
*Agents acting on the renin-angiotensin system*	captopril, losartan
*Anaesthetics*	lidocaine
*Psycholeptics*	olanzapine
*Psychoanaleptics*	fluoxetine
*Other nervous system drugs*	nicotine

Psoriasis studies are available for numerous potential drugs with high centralities. “Blood glucose lowering drugs” are promising drug candidates. The biguanide metformin is associated with reduced psoriasis risk in a population based case control study.[46] Many studies are available about “Thiazolidinedione” group. A recent meta-analysis showed significant decrease in Psoriasis Area and Severity Index (PASI) scores compared to placebo in case of pioglitazone and non-significant improvement in PASI 50/70 in case of rosiglitazone.[47] Troglitazone normalized histological features in psoriasis models and the lesional phenotype in a small clinical trial.[48] The “HMG CoA reductase inhibitor” drug simvastatin was effective in a pilot study, although atorvastatin in the same class showed only a non-significant improvement in a different study.[49], [50] Salicylic acid has antifungal effects and it's used as adjuvant because of its keratolytic effect in the treatment of psoriasis.[51] The “Antineoplastic agent” methotrexate is a well-known medication for psoriasis but several additional drugs in the same class were found in our analysis. Studies are available about 5-fluorouracil for the treatment of dystrophic psoriatic fingernails, but it showed only non-significant improvement.[52] Micellar paclitaxel significantly improved psoriasis in a prospective phase II study.[53] A study reported significant effectiveness of topical caffeine.[54] “Calcium channel blocker” nifedipine is found to be inductor of the disease in a case control study.[55] A study in 2005 reported significant PASI score reduction of 49.9% by topical theophylline ointment.[56] Mahonia aquafolium extract - consisting of berberine among others - is not classified into ATC classes, but three clinical trials already indicated improvement of psoriasis with this substance.[57] Multiple studies prove efficacy of the terpenoid triptolide in the treatment of psoriasis.[58] A recent study investigated effect of rifampicin on psoriasis and reported a 50.03% mean PASI reduction.[59] Study about the treatment of psoriasis with curcumin was carried out but reported only low response rate.[60]

In an in vitro experiment the “Lipid modifying agent” clofibrate, but not bezafibrate reversed UVB-light-mediated expression of psoriasis – related inflammatory cytokines (interleukin-6, interleukin-8).[61] Fluvastatin and pravastatin have the potential to inhibit Th17 cell chemotaxis thus lowering immune cell infiltration of psoriatic skin.[62] Anti-proliferative effect of novel COX2 inhibitors on HaCaT keratinocytes was proven in an in vitro experiment and possible therapeutic use in psoriasis was supposed. However no such experiment was carried out with celecoxib which was the only COX2 inhibitor in best rated drugs.[63] N-acetyl-cysteine attenuated TNF alpha – induced cytokine production in primary human keratinocytes, which suggests its anti-psoriatic potential.[64] The “Thiazolidinedione” ciglitazone was never used as a medication, but inhibited keratinocyte proliferation in a dose dependent fashion.[48] Histone – deacetylase inhibitor trichostatin A blocked the conversion of regulatory T cells to IL17 expressing T cells suggesting its beneficial role in treating psoriasis.[65] Tse et al. suppose that antiproliferative effect of arsenic compounds could have positive effects on psoriatic skin.[66] The phosphodiesterase inhibitor rolipram has the ability to block enterotoxin B-mediated induction of skin homing receptor on T lymphocytes and may have the potential to inhibit lymphocytic infiltration of lesional skin.[67] The natural polyphenolic compound rottlerin is a potent inhibitor of NFκB and may have disease modulating effects.[68]

Case reports are available about psoriasis induction by clonidine, “agents acting on the renin-angiotensin system” like captopril or losartan; the “protein kinase inhibitor” and “antineoplastic agent” imatinib; diclofenac, olanzapine, fluoxetine and chloroquine. Also case reports are available about the beneficial effects of ritonavir; “antineoplastic agents” like cytarabine, doxorubicin, and cysplatin; gefitinib, colchicine, lidocaine and nicotine.[69]–[83]

**Figure 5:**
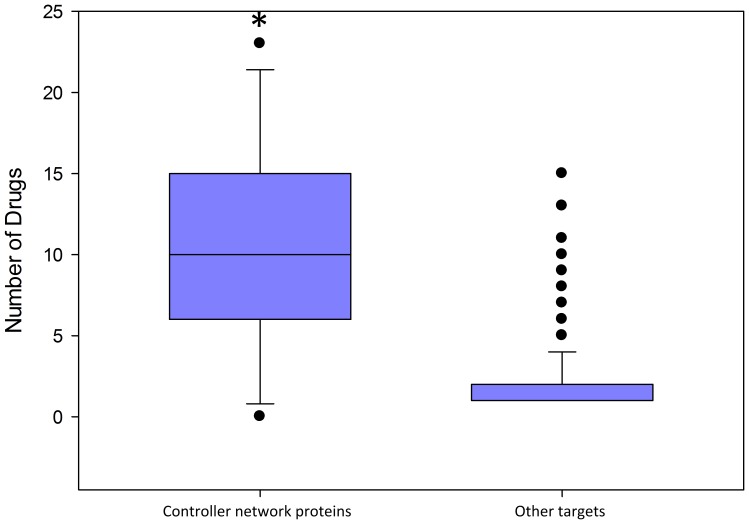
Effect of anti-psoriatic drugs on controller network. Higher number of effective anti-psoriatic drugs act on controller nodes than on other proteins. Totally the targets of 32 effective anti-psoriatic drugs were analyzed (median 10 vs. 1) *p<0.001

The 32 effective drugs of “Studies available” group in Table 7 were filtered out from STITCH data and target proteins were analyzed. All target proteins got an in-degree value reflecting the number of effective drugs acting on it. The group of proteins forming the controller sub-network was compared with the remaining target proteins. The controller sub-network protein group got significantly higher median value (10 vs. 1) using Mann-Whitney Rank Sum Test than the other one, which prove the importance of the controller sub-network in psoriatic lesions. (Figure 5) (p<0.001; in-degree has power law distribution, thus T-test could not be used) Higher median value could be caused by higher original degree centralities of controller network proteins in PPI networks, but only weak relation have been found between original degree centrality and the number of effective drugs acting on a protein, which cannot explain the big difference between the median of two groups (corrected R square value in regression analysis: 0.304)

In summary, studies are available for 34 drugs found by our analysis, experimental evidence is available for 24 drugs, case reports suggest beneficial or disease-inductor effect of 21 drugs and 98 unpublished drug candidates for the treatment of psoriasis were also found (Table 7–8).

**Table 8:** Drug candidates unassociated with psoriasis.

ATC Class	Drug
*Retinoids for topical use in acne*	retinol
*Blood glucose lowering drugs excl. insulines*	glyburide
*Vitamin K and other hemostatics*	menadione
*Antineoplastic agents*	aldophosphamide, MLS003389283, etoposide, dasatinib, decitabine
*Sex hormones and modulators of the genital system*	(4–14c)pregn-4-ene-3,20-dione, mifepristone, testosterone-propionate, androstanolone, diethylstilbestrol, raloxifene
*Hormone antagonists and related agents*	flutamide, fulvestrant
*Cardiac stimulants excl. cardiac glycosides*	bucladesine
*Cardiac glycosides*	G-Strophantin
*Drugs for obstructive airway diseases*	salbutamol
*Antiadrenergic agents, centrally acting*	reserpine
*Antiadrenergic agents, peripherally acting*	prazosin
*Lipid modifying agents, plain*	lovastatin, pitavastatin, fenofibrate
*Calcium channel blockers*	verapamil
*Diuretics*	furosemide, spironolactone
*Liver therapy*	silibinin
*Platelet aggregation inhibitors excl. heparin*	dipyridamole, cilostazol, amiloride-hydrochloride
*Agents acting on the renin-angiotensin system*	telmisartan, valsartan
*Anaesthetics*	ketamine, propofol, cocaine, isoflurane
*Analgesics*	morphine
*Psycholeptics*	haloperidol, clozapine, diazepam
*Psychoanaleptics*	desipramine, amitriptyline, metamphetamine
*Antiepileptics*	phenobarbital, valproic acid
*Antidotes*	naloxone
*Other nervous system drugs*	carbacholin
*N/A*	cytochalasin D, aminoguanidine, Neurogard, paraquat, Y27632, oxidopamine, nitroarginine, AC1LA4H9, SL327, emodin, 2,3,7,8-tetrachlorodibenzo-dioxin, 3-(2-aminoethyl)-5-[(4-ethoxyphenyl)methylidene]-1,3-thiazolidine-2,4-dione, CHEMBL248238, geldanamycin, anisomycin, 8-bromocyclic GMP, tempol, MK-801, 1-(5-isoquinolinesulfonyl)-2-methylpiperazine, ionomycin, herbimycin, pyrrolidine dithiocarbamate, nordihydroguaiaretic acid, gamma-imino-ATP, forskolin, GMP-Pnp, roscovitine, flavopiridol, N-formyl-Met-Leu-Phe, ns-398, sodium butyrate, AC1L1I8V, tyrphostin B42, kainic acid, pirinixic acid, IBMX, bisindolmaleimide I, proline-dithiocarbamate, KBio2_002303, Zillal, thapsigargin, calcimycin, clenbuterol, indole-3-carbinol, 1,9-pyrazoloanthrone, herbimycin, kaempferol, daidzein, lithium-chloride, naringenin

## Discussion

### Microarray Meta-analysis

Previous meta-analysis of psoriasis microarray studies was carried out by Tian et al. 1120 DEGs were found using 5 studies and 1832 DEGs using 3 studies.[84] We used the same 5 studies, but samples with inadequate quality were excluded from each study using arrayQualityMetrics package. The high number of DEGs (5363) in our study may be surprising, but it can be caused by the lower gene expression fold change cutoff (1.5 and −1.5 instead of 2 and −2). The pre - filtering process of samples can decrease variance and can also increase the number of DEGs. Further analysis of DEGs was carried out with Ingenuity Pathway Analysis (IPA) by Tian et al. IPA uses published references, carry out gene set enrichment analysis and TF detection. We used fundamentally different analysis. We generated PPI networks based on the largest PPI database (STRING) available which not only contain experimentally proven interactions but highly reliable interactions based on prediction algorithms or data mining. PDI network was also generated using not only literally proven interactions but interactions based on high fidelity prediction algorithms. Using lower DEG fold-change cutoff and detailed analysis based on node centrality statistics made it possible to identify proteins yet not associated with the disease but may have remarkable impact on pathogenesis. A chemical – protein interaction network based on STITCH database was also created and disease – modifying drug prediction was also possible with this method.

### Keratinocyte hyperproliferation and Psoriasis

Keratinocyte hyperproliferation and inhibition of apoptosis are well-known phenomena in psoriasis. Several proteins have been associated with these mechanisms like BCL2, BAX, NFATC1, PPARδ, EGF, mTOR, NF-κB etc.[85]–[88] Most of them were in central proteins detected by DEG-derived network analysis. Candidate DEG-coded proteins for hyperproliferation like CCNA2, TFDP1 and MECOM were also found. CCNA2 encodes Cyclin A2, that controls S phase and G2/M transition. Not only cell cycle progression is abnormal in lesional skin, but actin cytoskeleton organization as well.[89] A recent study reported that CCNA2 protein has role in cytoskeletal rearrangements and cell migration as well.[31] Cyclin A2 may take part in hyperproliferation and in aberrant actin cytoskeleton organization in psoriatic skin keratinocytes. TFDP1 encodes DP1 protein which is a dimerization partner of E2F transcription factor. The E2F/DP1 heterodimers regulate cell cycle via DNA replication control and apoptosis. DP1 has E2F-independent function as well: DP1 can stabilize Wnt-on and Wnt-off states in Wnt/β-catenin signaling and determine differential cell fates.[37] TFDP1-regulated genes belong to cell cycle progression as shown by BinGO analysis (Table S5). TFDP1 also has a reciprocal gene expression regulation with AR. This interaction was responsible for motif no. 332 enrichment in psoriasis PDI network compared to complete PDI network. This interaction may connect the hyperproliferation machinery to the merged controller sub-network.

### Immunological-metabolic interplay in psoriasis

Psoriasis is an immune-mediated disease. Some proteins which are published as important factors in pathogenesis were absent from DEGs in our microarray-meta analysis, such as TNF alpha, which is an important target in psoriasis therapy. This could be explained by the fact, that increased TNF alpha in psoriatic plaques can be caused mainly by post-transcriptional mechanisms.[90]

Many proteins published in association with the immunopathogenesis of psoriasis were highly ranked hubs in PPI networks: IL1, IL8, TGFB1, SP1, STAT1, STAT3, NFKB1, IRF1 etc.[87], [91]–[97] A highly interconnected cluster mainly consisting of upregulated chemokines and chemokine receptors was also found by PPI analysis (Figure S3). The downregulation of src kinase FYN seems to be a counteracting compensatory mechanism as this protein is important in IFN gamma action, in TNF alpha induced COX2 expression and in adipose tissue - mediated inflammation leading to insulin resistance. These processes are important in the pathomechanism of psoriasis.[32], [98], [99] These data suggest that the FYN inhibitor KBio2_002303 may have beneficial effects in the treatment of psoriasis. An important node in controller sub-network is IL8. Although its role in psoriasis pathogenesis is published, no trial has been done with IL8 inhibitors.[100] This is true for CCL2 and IRF1 as well. Our study confirms their basic role in sustainment of lesional phenotype. Both can be found in highly ranked hubs and CCL2 is also essential in controller sub – network by activating two positive feedback loops related to inflammation.

Psoriasis and metabolic syndrome comorbidity is a well-known phenomenon. There is a complicated interaction between the two diseases mediated by inflammatory cytokines among others.[101] Numerous DEG-coded proteins associated with both diseases could be found in central proteins like PPARG, INS-IGF2, LEP etc.[102]–[104] Others, like PIK3R1, AR and MEF2A may have role in the development of metabolic syndrome in psoriasis. PI3KR1 is important in the development of insulin resistance, it propagates inflammatory response in obese mice and may be an important link between the obesity-inflammation interplay in psoriasis.[33] AR has important effect on insulin signaling and thus insulin resistance. It is published that AR knockout mice exhibit insulin resistance.[35] To our knowledge AR has not yet been associated with psoriasis. However it was found in 1981, that lower serum testosterone level therefore decreased AR activation can be detected in psoriatic patients.[105] AR and PPARG connect inflammation- and metabolism-related hubs in controller network thus modulation of these proteins can be beneficial in psoriatic patients, which was also proven by our drug target analysis (Figure 5). MEF2A is important for GLUT4 expression on insulin-responsive cells. Expression of MEF2A is downregulated in lesional skin samples which suggests a possible mechanism for insulin resistance in psoriasis.

Many drugs, which are already widely used as treatment for psoriasis could be found in highly ranked nodes of chemical-protein interaction networks such as methotrexate, retinoic acid, corticosteroids, sirolimus and tacrolimus. According to STITCH data all of them act through at least one of the hubs in controller sub-network. Top ranked ATC drug classes target members of controller sub-network as well. Blood glucose-lowering drugs act through PPARG and INS-IGF2 activation, which can be the basis of the positive effects of fibrate and HMG-CoA inhibitors in psoriasis as well.[47] Cardiac stimulants such as adrenergic agents also have high impact on lesional skin's PPI and PDI network, mainly by modulating hubs in controller sub-network. “Sex hormones and modulators of the genital system” ATC drug class act on AR. The “antineoplastic drug” methotrexate mainly acts through the accumulation of adenosine, but other antineoplastic agents may have their effect on keratinocyte hyperproliferation.[106] Studies or case reports already suggest efficacy of some antineoplastic drugs but several new possible agents were found in our analysis.[53], [107], [108] Mental stress is a known trigger for psoriasis and connection between the neuroendocrine system and skin immune system has been reported. [3], [109] This is not surprising that numerous drugs acting on the CNS are enriched in highly ranked drugs. A lot of other drugs which are either classified in ATC classes or just drug candidates are found like kainic acid, cocaine, the HDAC inhibitor sodium butyrate, the PKC inhibitor bisindolylmaleimide I etc. (Table 7)

In summary this is the first time PPI, PDI and chemical-protein interaction networks of psoriatic skin samples has been examined with detailed network analysis. Network-building DEGs were identified with fine-quality microarray meta-analysis of 187 non-lesional and 189 lesional samples. Several proteins were found which are yet not associated with psoriasis but may have high impact on the pathogenesis of the disease. Basic disease controller sub-network was also constructed consisting of central nodes coded by DEGs. Numerous anti-psoriatic drugs and drug candidates were also found acting mainly on these nodes.

## Supporting Information

Figure S1 **FYN protein in the jActiveModules cluster with 2^nd^ highest score.** Nodes with blue-shaded color are downregulated and nodes with red-shaded color are upregulated. Color intensity is proportional with fold change.
(PNG)

Figure S2 **PIK3R1 protein in the jActiveModules cluster with 3^rd^ highest score.** Nodes with blue-shaded color are downregulated and nodes with red-shaded color are upregulated. Color intensity is proportional with fold change.
(PNG)

Figure S3 **Chemokine-chemokine receptor cluster found by ClusterONE.**
(PNG)

Table S1 **Outlier samples in arrayQualityMetrics analysis and explanation of exclusion.** Numbers from 1 to 5 indicate the number of method used by the software to assess quality.
(XLSX)

Table S2 **Results of repeated original statistics.** The same statistics, cutoff and filtering was used as it is shown in table after arrayQualityMetrics sample filtering and GCRMA normalization.
(XLSX)

Table S3 **Study-level p values of T test for differential gene expression and meta-analysis FDR values.** DEGs are highlighted with orange color.
(XLSX)

Table S4 **Node centralities in each network.** Different networks can be found on different worksheets. Columns indicate centrality values calculated by NetworkAnalyzer. Central nodes are highlighted with orange color.
(XLSX)

Table S5 **Results of BinGO analysis.** Significant GO terms are highlighted with orange color
(XLSX)

Dataset S1 **Results of arrayQualityMetrics analysis.** Only html data can be found in directories, pdf files were deleted due to size restrictions.
(ZIP)

## References

[1] Parisi R, Symmons DP, Griffiths CE, Ashcroft DM (2013) Global epidemiology of psoriasis: a systematic review of incidence and prevalence. J Invest Dermatol 133: 377–385. 23014338 10.1038/jid.2012.339

[2] Tsoi LC, Spain SL, Knight J, Ellinghaus E, Stuart PE, et al (2012) Identification of 15 new psoriasis susceptibility loci highlights the role of innate immunity. Nat Genet 44: 1341–1348. 23143594 10.1038/ng.2467 PMC3510312

[3] Griffiths CE, Barker JN (2007) Pathogenesis and clinical features of psoriasis. Lancet 370: 263–271. 17658397 10.1016/S0140-6736(07)61128-3

[4] Farkas A, Kemeny L (2012) Monocyte-derived interferon-alpha primed dendritic cells in the pathogenesis of psoriasis: new pieces in the puzzle. Int Immunopharmacol 13: 215–218. 22522054 10.1016/j.intimp.2012.04.003

[5] Armstrong AW, Harskamp CT, Armstrong EJ (2013) Psoriasis and metabolic syndrome: A systematic review and meta-analysis of observational studies. J Am Acad Dermatol. 10.1016/j.jaad.2012.08.015 23360868

[6] van der Fits L, Mourits S, Voerman JS, Kant M, Boon L, et al (2009) Imiquimod-induced psoriasis-like skin inflammation in mice is mediated via the IL-23/IL-17 axis. J Immunol 182: 5836–5845. 19380832 10.4049/jimmunol.0802999

[7] Campain A, Yang YH (2010) Comparison study of microarray meta-analysis methods. BMC Bioinformatics 11: 408. 20678237 10.1186/1471-2105-11-408 PMC2922198

[8] Barabasi AL, Albert R (1999) Emergence of scaling in random networks. Science 286: 509–512. 10521342 10.1126/science.286.5439.509

[9] Brandes U, Erlebach T (2005) Network analysis: methodological foundations. Berlin; New York: Springer. xii, 471 p. p.

[10] Ingram PJ, Stumpf MP, Stark J (2006) Network motifs: structure does not determine function. BMC Genomics 7: 108. 16677373 10.1186/1471-2164-7-108 PMC1488845

[11] Kauffmann A, Gentleman R, Huber W (2009) arrayQualityMetrics–a bioconductor package for quality assessment of microarray data. Bioinformatics 25: 415–416. 19106121 10.1093/bioinformatics/btn647 PMC2639074

[12] Servant N, Gravier E, Gestraud P, Laurent C, Paccard C, et al (2010) EMA - A R package for Easy Microarray data analysis. BMC Res Notes 3: 277. 21047405 10.1186/1756-0500-3-277 PMC2987873

[13] Kang DD, Sibille E, Kaminski N, Tseng GC (2012) MetaQC: objective quality control and inclusion/exclusion criteria for genomic meta-analysis. Nucleic Acids Res 40: e15. 22116060 10.1093/nar/gkr1071 PMC3258120

[14] Subramanian A, Tamayo P, Mootha VK, Mukherjee S, Ebert BL, et al (2005) Gene set enrichment analysis: a knowledge-based approach for interpreting genome-wide expression profiles. Proc Natl Acad Sci U S A 102: 15545–15550. 16199517 10.1073/pnas.0506580102 PMC1239896

[15] Wang X, Kang DD, Shen K, Song C, Lu S, et al (2012) An R package suite for microarray meta-analysis in quality control, differentially expressed gene analysis and pathway enrichment detection. Bioinformatics 28: 2534–2536. 22863766 10.1093/bioinformatics/bts485 PMC3463115

[16] Derkach A, Lawless JF, Sun L (2013) Robust and powerful tests for rare variants using Fisher's method to combine evidence of association from two or more complementary tests. Genet Epidemiol 37: 110–121. 23032573 10.1002/gepi.21689

[17] Morgan AA, Khatri P, Jones RH, Sarwal MM, Butte AJ (2010) Comparison of multiplex meta analysis techniques for understanding the acute rejection of solid organ transplants. BMC Bioinformatics 11 Suppl 9S6. 10.1186/1471-2105-11-S9-S6 PMC2967747 21044364

[18] Szklarczyk D, Franceschini A, Kuhn M, Simonovic M, Roth A, et al (2011) The STRING database in 2011: functional interaction networks of proteins, globally integrated and scored. Nucleic Acids Res 39: D561–568. 21045058 10.1093/nar/gkq973 PMC3013807

[19] Kuhn M, Szklarczyk D, Franceschini A, von Mering C, Jensen LJ, et al (2012) STITCH 3: zooming in on protein-chemical interactions. Nucleic Acids Res 40: D876–880. 22075997 10.1093/nar/gkr1011 PMC3245073

[20] Robertson G, Bilenky M, Lin K, He A, Yuen W, et al (2006) cisRED: a database system for genome-scale computational discovery of regulatory elements. Nucleic Acids Res 34: D68–73. 16381958 10.1093/nar/gkj075 PMC1347438

[21] Wingender E, Dietze P, Karas H, Knuppel R (1996) TRANSFAC: a database on transcription factors and their DNA binding sites. Nucleic Acids Res 24: 238–241. 8594589 10.1093/nar/24.1.238 PMC145586

[22] Sandelin A, Alkema W, Engstrom P, Wasserman WW, Lenhard B (2004) JASPAR: an open-access database for eukaryotic transcription factor binding profiles. Nucleic Acids Res 32: D91–94. 14681366 10.1093/nar/gkh012 PMC308747

[23] Smoot ME, Ono K, Ruscheinski J, Wang PL, Ideker T (2011) Cytoscape 2.8: new features for data integration and network visualization. Bioinformatics 27: 431–432. 21149340 10.1093/bioinformatics/btq675 PMC3031041

[24] Newman M (2005) Power laws, Pareto distributions and Zipf's law. Contemporary Physics 46: 323–351.

[25] Li X, Stones DS, Wang H, Deng H, Liu X, et al (2012) NetMODE: network motif detection without Nauty. PLoS One 7: e50093. 23272055 10.1371/journal.pone.0050093 PMC3525646

[26] Ferro A, Giugno R, Pigola G, Pulvirenti A, Skripin D, et al (2007) NetMatch: a Cytoscape plugin for searching biological networks. Bioinformatics 23: 910–912. 17277332 10.1093/bioinformatics/btm032

[27] Nepusz T, Yu H, Paccanaro A (2012) Detecting overlapping protein complexes in protein-protein interaction networks. Nat Methods 9: 471–472. 22426491 10.1038/nmeth.1938 PMC3543700

[28] Cline MS, Smoot M, Cerami E, Kuchinsky A, Landys N, et al (2007) Integration of biological networks and gene expression data using Cytoscape. Nat Protoc 2: 2366–2382. 17947979 10.1038/nprot.2007.324 PMC3685583

[29] Maere S, Heymans K, Kuiper M (2005) BiNGO: a Cytoscape plugin to assess overrepresentation of gene ontology categories in biological networks. Bioinformatics 21: 3448–3449. 15972284 10.1093/bioinformatics/bti551

[30] Lu X, Jain VV, Finn PW, Perkins DL (2007) Hubs in biological interaction networks exhibit low changes in expression in experimental asthma. Mol Syst Biol 3: 98. 17437023 10.1038/msb4100138 PMC1865580

[31] Arsic N, Bendris N, Peter M, Begon-Pescia C, Rebouissou C, et al (2012) A novel function for Cyclin A2: control of cell invasion via RhoA signaling. J Cell Biol 196: 147–162. 22232705 10.1083/jcb.201102085 PMC3255987

[32] Smyth D, Phan V, Wang A, McKay DM (2011) Interferon-gamma-induced increases in intestinal epithelial macromolecular permeability requires the Src kinase Fyn. Lab Invest 91: 764–777. 21321534 10.1038/labinvest.2010.208

[33] McCurdy CE, Schenk S, Holliday MJ, Philp A, Houck JA, et al (2012) Attenuated Pik3r1 expression prevents insulin resistance and adipose tissue macrophage accumulation in diet-induced obese mice. Diabetes 61: 2495–2505. 22698915 10.2337/db11-1433 PMC3447911

[34] Laug R, Fehrholz M, Schutze N, Kramer BW, Krump-Konvalinkova V, et al (2012) IFN-gamma and TNF-alpha synergize to inhibit CTGF expression in human lung endothelial cells. PLoS One 7: e45430. 23029004 10.1371/journal.pone.0045430 PMC3447888

[35] Lin HY, Yu IC, Wang RS, Chen YT, Liu NC, et al (2008) Increased hepatic steatosis and insulin resistance in mice lacking hepatic androgen receptor. Hepatology 47: 1924–1935. 18449947 10.1002/hep.22252

[36] Lai JJ, Lai KP, Zeng W, Chuang KH, Altuwaijri S, et al (2012) Androgen receptor influences on body defense system via modulation of innate and adaptive immune systems: lessons from conditional AR knockout mice. Am J Pathol 181: 1504–1512. 22959669 10.1016/j.ajpath.2012.07.008 PMC3483803

[37] Kim WT, Kim H, Katanaev VL, Joon Lee S, Ishitani T, et al (2012) Dual functions of DP1 promote biphasic Wnt-on and Wnt-off states during anteroposterior neural patterning. EMBO J 31: 3384–3397. 22773187 10.1038/emboj.2012.181 PMC3419924

[38] Roy S, Jorgensen HG, Roy P, Abed El Baky M, Melo JV, et al (2012) BCR-ABL1 tyrosine kinase sustained MECOM expression in chronic myeloid leukaemia. Br J Haematol 157: 446–456. 22372463 10.1111/j.1365-2141.2012.09078.x

[39] Mora S, Pessin JE (2000) The MEF2A isoform is required for striated muscle-specific expression of the insulin-responsive GLUT4 glucose transporter. J Biol Chem 275: 16323–16328. 10748204 10.1074/jbc.M910259199

[40] Ma'ayan A, Lipshtat A, Iyengar R, Sontag ED (2008) Proximity of intracellular regulatory networks to monotone systems. IET Syst Biol 2: 103–112. 18537452 10.1049/iet-syb:20070036 PMC2453221

[41] Beutler B (2009) Microbe sensing, positive feedback loops, and the pathogenesis of inflammatory diseases. Immunol Rev 227: 248–263. 19120489 10.1111/j.1600-065X.2008.00733.x PMC2713013

[42] Brandman O, Ferrell JE Jr, Li R, Meyer T (2005) Interlinked fast and slow positive feedback loops drive reliable cell decisions. Science 310: 496–498. 16239477 10.1126/science.1113834 PMC3175767

[43] Miller GC, Britt H (1995) A new drug classification for computer systems: the ATC extension code. Int J Biomed Comput 40: 121–124. 8847119 10.1016/0020-7101(95)01135-2

[44] Kanehisa M, Goto S, Hattori M, Aoki-Kinoshita KF, Itoh M, et al (2006) From genomics to chemical genomics: new developments in KEGG. Nucleic Acids Res 34: D354–357. 16381885 10.1093/nar/gkj102 PMC1347464

[45] Menter A, Griffiths CE (2007) Current and future management of psoriasis. Lancet 370: 272–284. 17658398 10.1016/S0140-6736(07)61129-5

[46] Brauchli YB, Jick SS, Curtin F, Meier CR (2008) Association between use of thiazolidinediones or other oral antidiabetics and psoriasis: A population based case-control study. J Am Acad Dermatol 58: 421–429. 18194825 10.1016/j.jaad.2007.11.023

[47] Malhotra A, Shafiq N, Rajagopalan S, Dogra S, Malhotra S (2012) Thiazolidinediones for plaque psoriasis: a systematic review and meta-analysis. Evid Based Med 17: 171–176. 22522793 10.1136/ebmed-2011-100388

[48] Ellis CN, Varani J, Fisher GJ, Zeigler ME, Pershadsingh HA, et al (2000) Troglitazone improves psoriasis and normalizes models of proliferative skin disease: ligands for peroxisome proliferator-activated receptor-gamma inhibit keratinocyte proliferation. Arch Dermatol 136: 609–616. 10815854 10.1001/archderm.136.5.609

[49] Shirinsky IV, Shirinsky VS (2007) Efficacy of simvastatin in plaque psoriasis: A pilot study. J Am Acad Dermatol 57: 529–531. 17707157 10.1016/j.jaad.2007.05.040

[50] Faghihi T, Radfar M, Mehrabian Z, Ehsani AH, Rezaei Hemami M (2011) Atorvastatin for the treatment of plaque-type psoriasis. Pharmacotherapy 31: 1045–1050. 22026392 10.1592/phco.31.11.1045

[51] Lebwohl M (1999) The role of salicylic acid in the treatment of psoriasis. Int J Dermatol 38: 16–24. 10065604 10.1046/j.1365-4362.1999.00500.x

[52] de Jong EM, Menke HE, van Praag MC, van De Kerkhof PC (1999) Dystrophic psoriatic fingernails treated with 1% 5-fluorouracil in a nail penetration-enhancing vehicle: a double-blind study. Dermatology 199: 313–318. 10640840 10.1159/000018281

[53] Ehrlich A, Booher S, Becerra Y, Borris DL, Figg WD, et al (2004) Micellar paclitaxel improves severe psoriasis in a prospective phase II pilot study. J Am Acad Dermatol 50: 533–540. 15034502 10.1016/j.jaad.2003.09.018

[54] Vali A, Asilian A, Khalesi E, Khoddami L, Shahtalebi M, et al (2005) Evaluation of the efficacy of topical caffeine in the treatment of psoriasis vulgaris. J Dermatolog Treat 16: 234–237. 16249145 10.1080/09546630510011801

[55] Cohen AD, Kagen M, Friger M, Halevy S (2001) Calcium channel blockers intake and psoriasis: a case-control study. Acta Derm Venereol 81: 347–349. 11800142 10.1080/000155501317140061

[56] Papakostantinou E, Xenos K, Markantonis SL, Druska S, Stratigos A, et al (2005) Efficacy of 2 weeks' application of theophylline ointment in psoriasis vulgaris. J Dermatolog Treat 16: 169–170. 16096184 10.1080/09546630510043202

[57] Gulliver WP, Donsky HJ (2005) A report on three recent clinical trials using Mahonia aquifolium 10% topical cream and a review of the worldwide clinical experience with Mahonia aquifolium for the treatment of plaque psoriasis. Am J Ther 12: 398–406. 16148424 10.1097/01.mjt.0000174350.82270.da

[58] Han R, Rostami-Yazdi M, Gerdes S, Mrowietz U (2012) Triptolide in the treatment of psoriasis and other immune-mediated inflammatory diseases. Br J Clin Pharmacol 74: 424–436. 22348323 10.1111/j.1365-2125.2012.04221.x PMC3477344

[59] Tsankov N, Grozdev I (2011) Rifampicin–a mild immunosuppressive agent for psoriasis. J Dermatolog Treat 22: 62–64. 20653488 10.3109/09546630903496975

[60] Kurd SK, Smith N, VanVoorhees A, Troxel AB, Badmaev V, et al (2008) Oral curcumin in the treatment of moderate to severe psoriasis vulgaris: A prospective clinical trial. J Am Acad Dermatol 58: 625–631. 18249471 10.1016/j.jaad.2007.12.035 PMC4131208

[61] Kippenberger S, Loitsch SM, Grundmann-Kollmann M, Simon S, Dang TA, et al (2001) Activators of peroxisome proliferator-activated receptors protect human skin from ultraviolet-B-light-induced inflammation. J Invest Dermatol 117: 1430–1436. 11886504 10.1046/j.0022-202x.2001.01537.x

[62] Kim TG, Byamba D, Wu WH, Lee MG (2011) Statins inhibit chemotactic interaction between CCL20 and CCR6 in vitro: possible relevance to psoriasis treatment. Exp Dermatol 20: 855–857. 21824198 10.1111/j.1600-0625.2011.01343.x

[63] Sticozzi C, Belmonte G, Cervellati F, Di Capua A, Maioli E, et al (2013) Antiproliferative effect of two novel COX-2 inhibitors on human keratinocytes. Eur J Pharm Sci 49: 133–141. 23454135 10.1016/j.ejps.2013.02.009

[64] Young CN, Koepke JI, Terlecky LJ, Borkin MS, Boyd Savoy L, et al (2008) Reactive oxygen species in tumor necrosis factor-alpha-activated primary human keratinocytes: implications for psoriasis and inflammatory skin disease. J Invest Dermatol 128: 2606–2614. 18463678 10.1038/jid.2008.122 PMC4102307

[65] Soler DC, McCormick TS (2011) The dark side of regulatory T cells in psoriasis. J Invest Dermatol 131: 1785–1786. 21844929 10.1038/jid.2011.200 PMC3366427

[66] Tse WP, Cheng CH, Che CT, Lin ZX (2008) Arsenic trioxide, arsenic pentoxide, and arsenic iodide inhibit human keratinocyte proliferation through the induction of apoptosis. J Pharmacol Exp Ther 326: 388–394. 18451318 10.1124/jpet.107.134080

[67] Santamaria LF, Torres R, Gimenez-Arnau AM, Gimenez-Camarasa JM, Ryder H, et al (1999) Rolipram inhibits staphylococcal enterotoxin B-mediated induction of the human skin-homing receptor on T lymphocytes. J Invest Dermatol 113: 82–86. 10417623 10.1046/j.1523-1747.1999.00639.x

[68] Maioli E, Valacchi G (2010) Rottlerin: bases for a possible usage in psoriasis. Curr Drug Metab 11: 425–430. 20540694 10.2174/138920010791526097

[69] Ikai K (1995) Exacerbation and induction of psoriasis by angiotensin-converting enzyme inhibitors. J Am Acad Dermatol 32: 819. 10.1016/0190-9622(95)91487-0 7722036

[70] Lamba G, Palaniswamy C, Singh T, Shah D, Lal S, et al (2011) Psoriasis induced by losartan therapy: a case report and review of the literature. Am J Ther 18: e78–80. 20027103 10.1097/MJT.0b013e3181c6c0c2

[71] Woo SM, Huh CH, Park KC, Youn SW (2007) Exacerbation of psoriasis in a chronic myelogenous leukemia patient treated with imatinib. J Dermatol 34: 724–726. 17908148 10.1111/j.1346-8138.2007.00369.x

[72] Sendagorta E, Allegue F, Rocamora A, Ledo A (1987) Generalized pustular psoriasis precipitated by diclofenac and indomethacin. Dermatologica 175: 300–301. 3691905 10.1159/000248839

[73] Latini A, Carducci M (2003) Psoriasis during therapy with olanzapine. Eur J Dermatol 13: 404–405. 12948926

[74] Tan Pei Lin L, Kwek SK (2010) Onset of psoriasis during therapy with fluoxetine. Gen Hosp Psychiatry 32: 446 e449–446 e410. 10.1016/j.genhosppsych.2009.08.008 20633754

[75] Schopf RE, Ockenfels HM, Schultewolter T, Morsches B (1993) Chloroquine stimulates the mitogen-driven lymphocyte proliferation in patients with psoriasis. Dermatology 187: 100–103. 8358095 10.1159/000247215

[76] Chiricozzi A, Saraceno R, Cannizzaro MV, Nistico SP, Chimenti S, et al (2012) Complete resolution of erythrodermic psoriasis in an HIV and HCV patient unresponsive to antipsoriatic treatments after highly active antiretroviral therapy (Ritonavir, Atazanavir, Emtricitabine, Tenofovir). Dermatology 225: 333–337. 23295963 10.1159/000345762

[77] Pisano C, Tambaro R, Di Maio M, Formato R, Iaffaioli VR, et al (2004) Complete resolution of psoriasis in a patient treated with stealth liposomal doxorubicin and carboplatin for ovarian cancer. Arch Dermatol Res 296: 141–142. 15278368 10.1007/s00403-004-0489-9

[78] Paslin D (1990) Psoriasis without neutrophils. Int J Dermatol 29: 37–40. 2329025 10.1111/j.1365-4362.1990.tb03753.x

[79] Cagiano R, Bera I, Vermesan D, Flace P, Sabatini R, et al (2008) Psoriasis disappearance after the first phase of an oncologic treatment: a serendipity case report. Clin Ter 159: 421–425. 19169602

[80] Halverstam CP, Lebwohl M (2008) Nonstandard and off-label therapies for psoriasis. Clin Dermatol 26: 546–553. 18755374 10.1016/j.clindermatol.2007.10.023

[81] Perlman HH (1972) Remission of psoriasis vulgaris from the use of nerve-blocking agents. Arch Dermatol 105: 128–129. 10.1001/archderm.1972.01620040088028 5009614

[82] Staples J, Klein D (2012) Can nicotine use alleviate symptoms of psoriasis? Can Fam Physician 58: 404–408. 22611606 PMC3325452

[83] Zorzou MP, Stratigos A, Efstathiou E, Bamias A (2004) Exacerbation of psoriasis after treatment with an EGFR tyrosine kinase inhibitor. Acta Derm Venereol 84: 308–309. 15339078 10.1080/00015550410024634

[84] Tian S, Krueger JG, Li K, Jabbari A, Brodmerkel C, et al (2012) Meta-analysis derived (MAD) transcriptome of psoriasis defines the “core” pathogenesis of disease. PLoS One 7: e44274. 22957057 10.1371/journal.pone.0044274 PMC3434204

[85] Romanowska M, al Yacoub N, Seidel H, Donandt S, Gerken H, et al (2008) PPARdelta enhances keratinocyte proliferation in psoriasis and induces heparin-binding EGF-like growth factor. J Invest Dermatol 128: 110–124. 17637826 10.1038/sj.jid.5700943

[86] Buerger C, Malisiewicz B, Eiser A, Hardt K, Boehncke WH (2013) mTOR and its downstream signalling components are activated in psoriatic skin. Br J Dermatol. 10.1111/bjd.12271 23398394

[87] Goldminz AM, Au SC, Kim N, Gottlieb AB, Lizzul PF (2013) NF-kappaB: An essential transcription factor in psoriasis. J Dermatol Sci 69: 89–94. 23219896 10.1016/j.jdermsci.2012.11.002

[88] Hampton PJ, Jans R, Flockhart RJ, Parker G, Reynolds NJ (2012) Lithium regulates keratinocyte proliferation via glycogen synthase kinase 3 and NFAT2 (nuclear factor of activated T cells 2). J Cell Physiol 227: 1529–1537. 21678407 10.1002/jcp.22872 PMC4150531

[89] Choi JH, Choi DK, Sohn KC, Kwak SS, Suk J, et al (2012) Absence of a human DnaJ protein hTid-1S correlates with aberrant actin cytoskeleton organization in lesional psoriatic skin. J Biol Chem 287: 25954–25963. 22692211 10.1074/jbc.M111.313809 PMC3406679

[90] Johansen C, Funding AT, Otkjaer K, Kragballe K, Jensen UB, et al (2006) Protein expression of TNF-alpha in psoriatic skin is regulated at a posttranscriptional level by MAPK-activated protein kinase 2. J Immunol 176: 1431–1438. 16424170 10.4049/jimmunol.176.3.1431

[91] Buerger C, Richter B, Woth K, Salgo R, Malisiewicz B, et al (2012) Interleukin-1beta interferes with epidermal homeostasis through induction of insulin resistance: implications for psoriasis pathogenesis. J Invest Dermatol 132: 2206–2214. 22513786 10.1038/jid.2012.123

[92] Jordan CT, Cao L, Roberson ED, Pierson KC, Yang CF, et al (2012) PSORS2 is due to mutations in CARD14. Am J Hum Genet 90: 784–795. 22521418 10.1016/j.ajhg.2012.03.012 PMC3376640

[93] Kallimanis PG, Xenos K, Markantonis SL, Stavropoulos P, Margaroni G, et al (2009) Serum levels of transforming growth factor-beta1 in patients with mild psoriasis vulgaris and effect of treatment with biological drugs. Clin Exp Dermatol 34: 582–586. 19094132 10.1111/j.1365-2230.2008.03026.x

[94] Madonna S, Scarponi C, Sestito R, Pallotta S, Cavani A, et al (2010) The IFN-gamma-dependent suppressor of cytokine signaling 1 promoter activity is positively regulated by IFN regulatory factor-1 and Sp1 but repressed by growth factor independence-1b and Kruppel-like factor-4, and it is dysregulated in psoriatic keratinocytes. J Immunol 185: 2467–2481. 20644166 10.4049/jimmunol.1001426

[95] Hald A, Andres RM, Salskov-Iversen ML, Kjellerup RB, Iversen L, et al (2013) STAT1 expression and activation is increased in lesional psoriatic skin. Br J Dermatol 168: 302–310. 23013371 10.1111/bjd.12049

[96] Miyoshi K, Takaishi M, Nakajima K, Ikeda M, Kanda T, et al (2011) Stat3 as a therapeutic target for the treatment of psoriasis: a clinical feasibility study with STA-21, a Stat3 inhibitor. J Invest Dermatol 131: 108–117. 20811392 10.1038/jid.2010.255

[97] Jackson M, Howie SE, Weller R, Sabin E, Hunter JA, et al (1999) Psoriatic keratinocytes show reduced IRF-1 and STAT-1alpha activation in response to gamma-IFN. FASEB J 13: 495–502. 10064616 10.1096/fasebj.13.3.495

[98] Lee TW, Kwon H, Zong H, Yamada E, Vatish M, et al.. (2013) Fyn Deficiency Promotes a Preferential Increase in Subcutaneous Adipose Tissue Mass and Decreased Visceral Adipose Tissue Inflammation. Diabetes. 10.2337/db12-0920 PMC3636609 23321073

[99] Hwang MK, Kang NJ, Heo YS, Lee KW, Lee HJ (2009) Fyn kinase is a direct molecular target of delphinidin for the inhibition of cyclooxygenase-2 expression induced by tumor necrosis factor-alpha. Biochem Pharmacol 77: 1213–1222. 19174152 10.1016/j.bcp.2008.12.021

[100] Giustizieri ML, Mascia F, Frezzolini A, De Pita O, Chinni LM, et al (2001) Keratinocytes from patients with atopic dermatitis and psoriasis show a distinct chemokine production profile in response to T cell-derived cytokines. J Allergy Clin Immunol 107: 871–877. 11344355 10.1067/mai.2001.114707

[101] Davidovici BB, Sattar N, Prinz J, Puig L, Emery P, et al (2010) Psoriasis and systemic inflammatory diseases: potential mechanistic links between skin disease and co-morbid conditions. J Invest Dermatol 130: 1785–1796. 20445552 10.1038/jid.2010.103

[102] Senturk N, Aydin F, Birinci A, Yuksel EP, Kara N, et al (2008) Investigation for the leptin 1 and LEP G2548A gene polymorphism in psoriasis. Eur J Dermatol 18: 343–344. 18474470 10.1684/ejd.2008.0403

[103] Michalik L, Wahli W (2007) Peroxisome proliferator-activated receptors (PPARs) in skin health, repair and disease. Biochim Biophys Acta 1771: 991–998. 17400022 10.1016/j.bbalip.2007.02.004

[104] Kwon YW, Jang ER, Lee YM, Kim YS, Kwon KS, et al (2000) Insulin-like growth factor II induces interleukin-6 expression via NFkappaB activation in psoriasis. Biochem Biophys Res Commun 278: 312–317. 11097836 10.1006/bbrc.2000.3806

[105] Schwarz W, Schell H, Hornstein OP (1981) Testosterone serum levels in male psoriatics. Arch Dermatol Res 270: 377–379. 6455973 10.1007/BF00403945

[106] Johnston A, Gudjonsson JE, Sigmundsdottir H, Ludviksson BR, Valdimarsson H (2005) The anti-inflammatory action of methotrexate is not mediated by lymphocyte apoptosis, but by the suppression of activation and adhesion molecules. Clin Immunol 114: 154–163. 15639649 10.1016/j.clim.2004.09.001

[107] Wang TS, Tsai TF (2012) Intralesional therapy for psoriasis. J Dermatolog Treat. 10.3109/09546634.2012.672706 22390539

[108] Kohn D, Flatau E, Daher O, Zuckerman F (1980) Treatment of psoriasis with daunorubicin and cytarabine. Arch Dermatol 116: 1101–1102. 6932826

[109] Scholzen T, Armstrong CA, Bunnett NW, Luger TA, Olerud JE, et al (1998) Neuropeptides in the skin: interactions between the neuroendocrine and the skin immune systems. Exp Dermatol 7: 81–96. 9583747 10.1111/j.1600-0625.1998.tb00307.x

[110] Gudjonsson JE, Ding J, Li X, Nair RP, Tejasvi T, et al (2009) Global gene expression analysis reveals evidence for decreased lipid biosynthesis and increased innate immunity in uninvolved psoriatic skin. J Invest Dermatol 129: 2795–2804. 19571819 10.1038/jid.2009.173 PMC2783967

[111] Yao Y, Richman L, Morehouse C, de los Reyes M, Higgs BW, et al (2008) Type I interferon: potential therapeutic target for psoriasis? PLoS One 3: e2737. 18648529 10.1371/journal.pone.0002737 PMC2481274

[112] (!!! INVALID CITATION !!!).

[113] Suarez-Farinas M, Li K, Fuentes-Duculan J, Hayden K, Brodmerkel C, et al (2012) Expanding the psoriasis disease profile: interrogation of the skin and serum of patients with moderate-to-severe psoriasis. J Invest Dermatol 132: 2552–2564. 22763790 10.1038/jid.2012.184 PMC3472561

[114] Reischl J, Schwenke S, Beekman JM, Mrowietz U, Sturzebecher S, et al (2007) Increased expression of Wnt5a in psoriatic plaques. J Invest Dermatol 127: 163–169. 16858420 10.1038/sj.jid.5700488

[115] Johnson-Huang LM, Pensabene CA, Shah KR, Pierson KC, Kikuchi T, et al (2012) Post-therapeutic relapse of psoriasis after CD11a blockade is associated with T cells and inflammatory myeloid DCs. PLoS One 7: e30308. 22348003 10.1371/journal.pone.0030308 PMC3277585

