# Supplementary figures and images for: Novel Factors in the Pathogenesis of Psoriasis and Potential Drug Candidates Are Found with Systems Biology Approach
Source: PLoS One. 2013 Nov 26;8(11):e80751. doi: 10.1371/journal.pone.0080751 (PMC3841158; doi:10.1371/journal.pone.0080751)

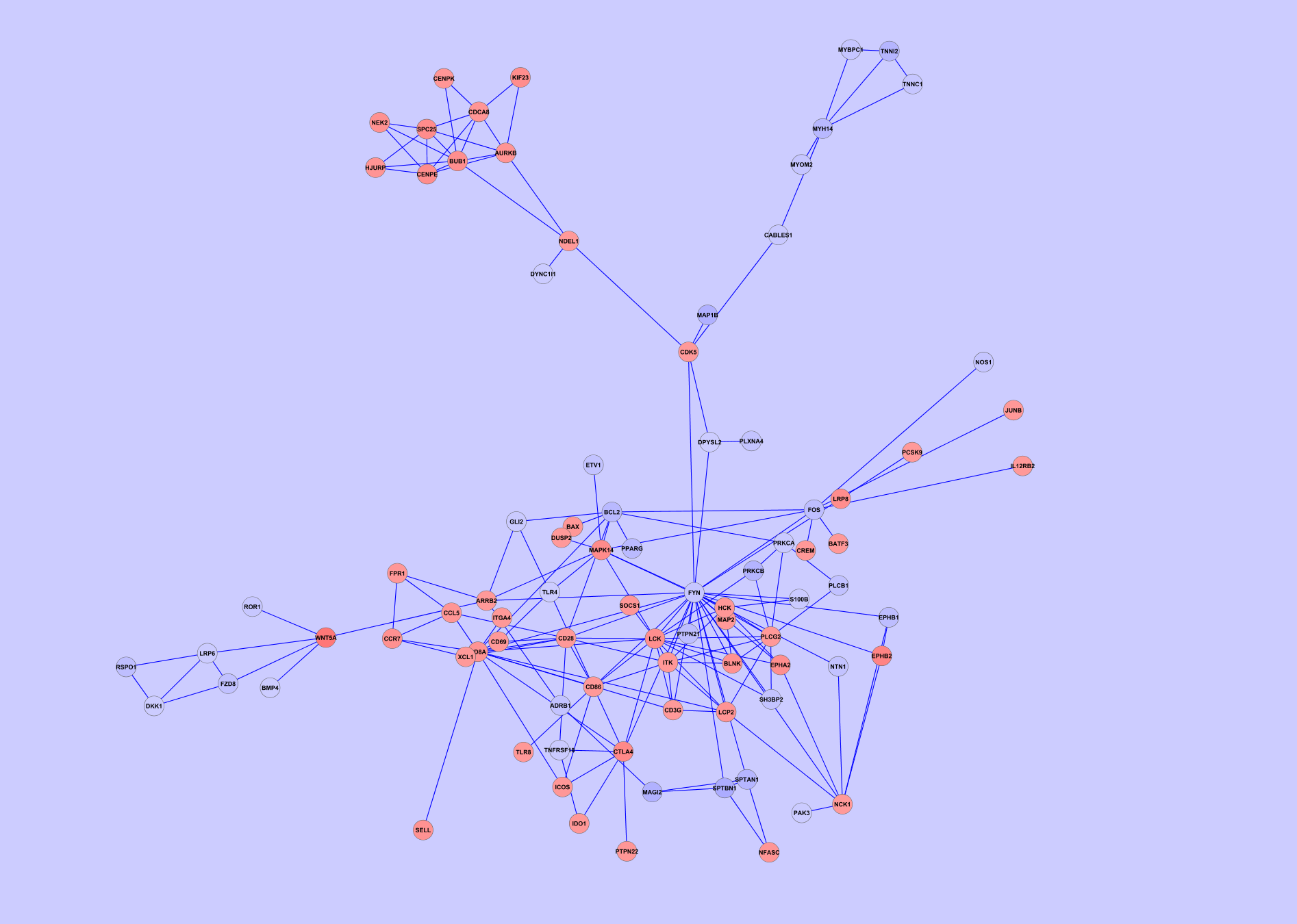

Supplement: Figure S1 — FYN protein in the jActiveModules cluster with 2nd highest score. Nodes with blue-shaded color are downregulated and nodes with red-shaded color are upregulated. Color intensity is proportional with fold change. (PNG) [file pone.0080751.s001.png]

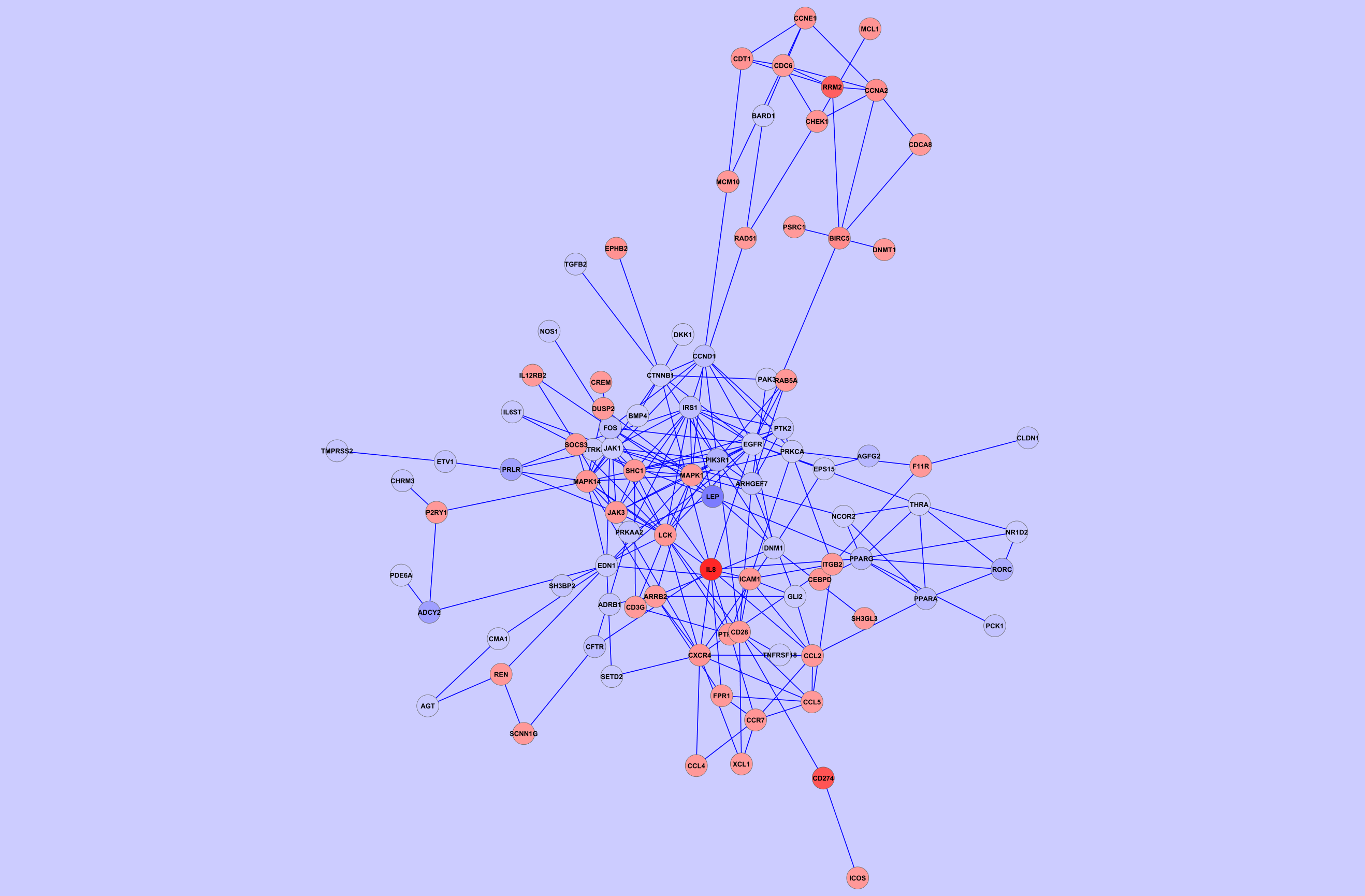

Supplement: Figure S2 — PIK3R1 protein in the jActiveModules cluster with 3rd highest score. Nodes with blue-shaded color are downregulated and nodes with red-shaded color are upregulated. Color intensity is proportional with fold change. (PNG) [file pone.0080751.s002.png]

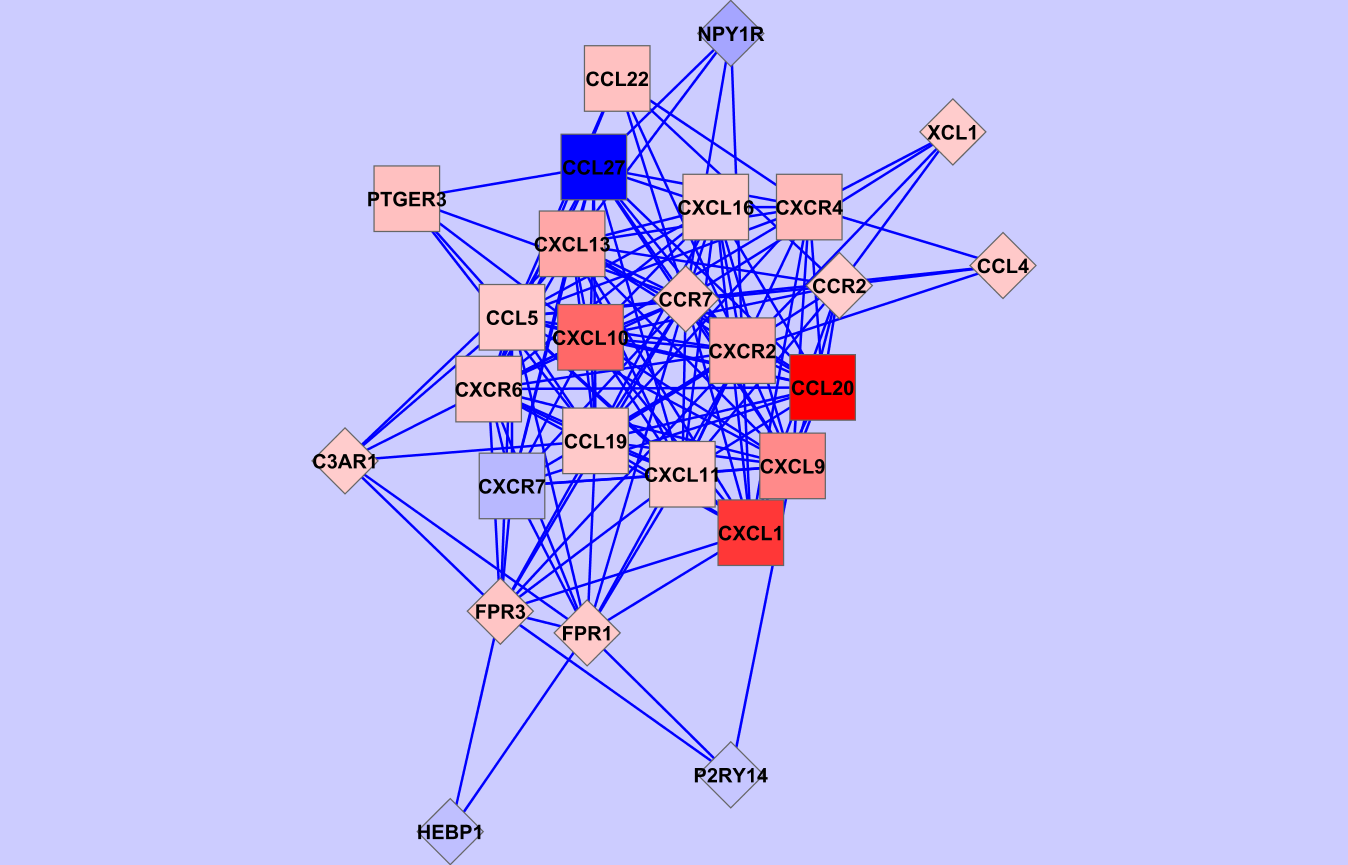

Supplement: Figure S3 — Chemokine-chemokine receptor cluster found by ClusterONE. (PNG) [file pone.0080751.s003.png]

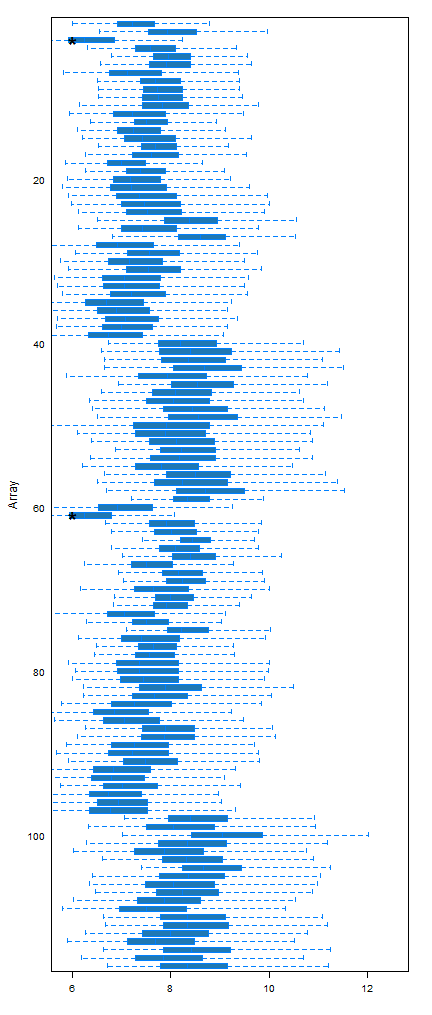

Supplement: Dataset S1 — Results of arrayQualityMetrics analysis. Only html data can be found in directories, pdf files were deleted due to size restrictions. (ZIP) [file pone.0080751.s009.zip › Supple/Gudjonsson et al/box.png]

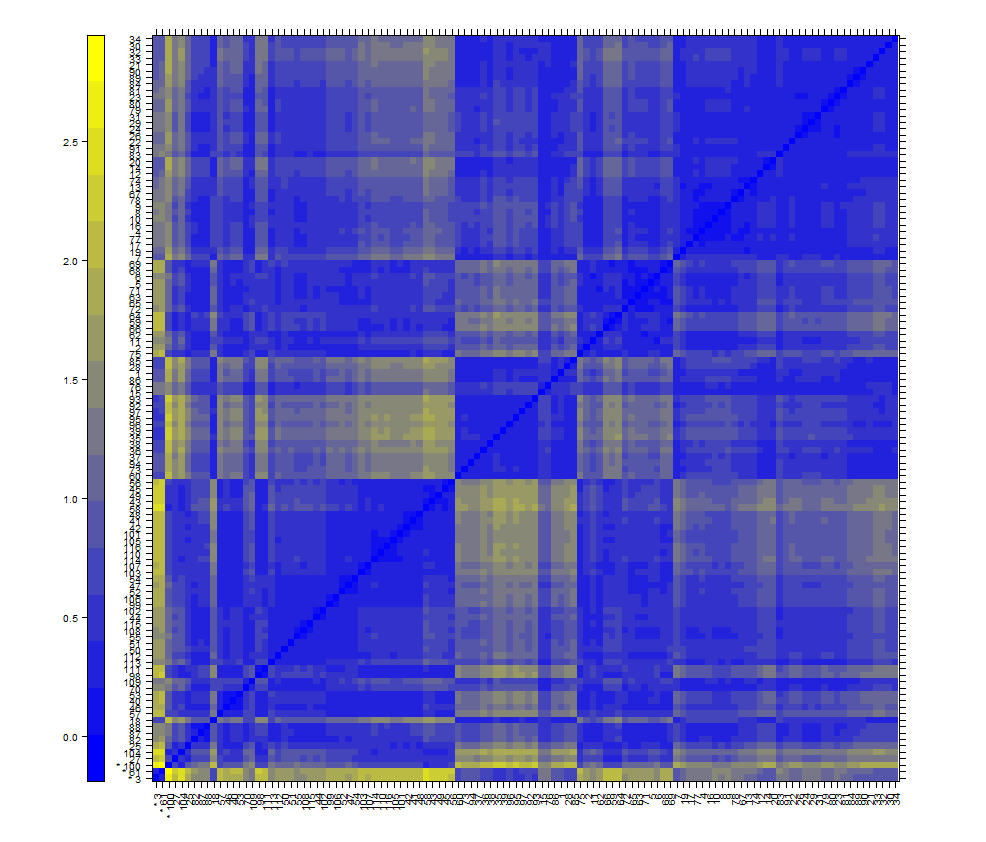

Supplement: Dataset S1 — Results of arrayQualityMetrics analysis. Only html data can be found in directories, pdf files were deleted due to size restrictions. (ZIP) [file pone.0080751.s009.zip › Supple/Gudjonsson et al/hm.png]

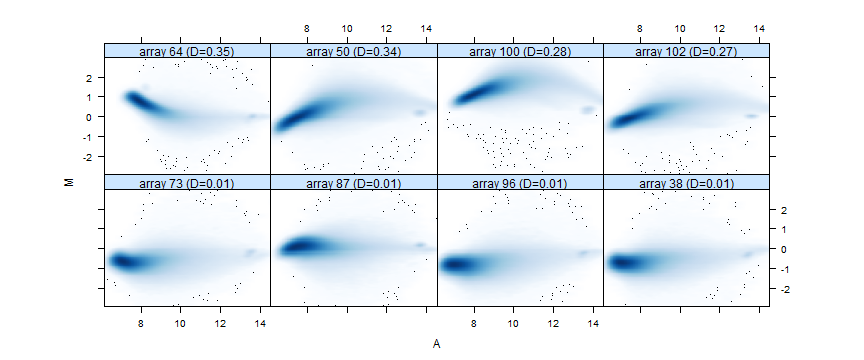

Supplement: Dataset S1 — Results of arrayQualityMetrics analysis. Only html data can be found in directories, pdf files were deleted due to size restrictions. (ZIP) [file pone.0080751.s009.zip › Supple/Gudjonsson et al/ma.png]

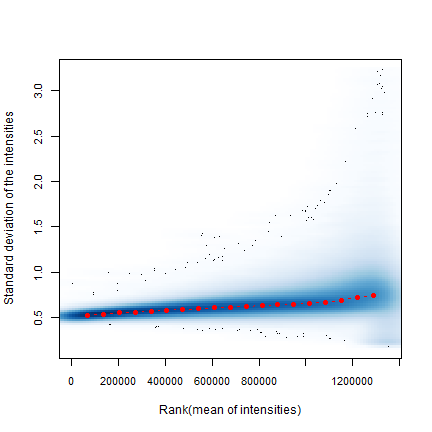

Supplement: Dataset S1 — Results of arrayQualityMetrics analysis. Only html data can be found in directories, pdf files were deleted due to size restrictions. (ZIP) [file pone.0080751.s009.zip › Supple/Gudjonsson et al/msd.png]

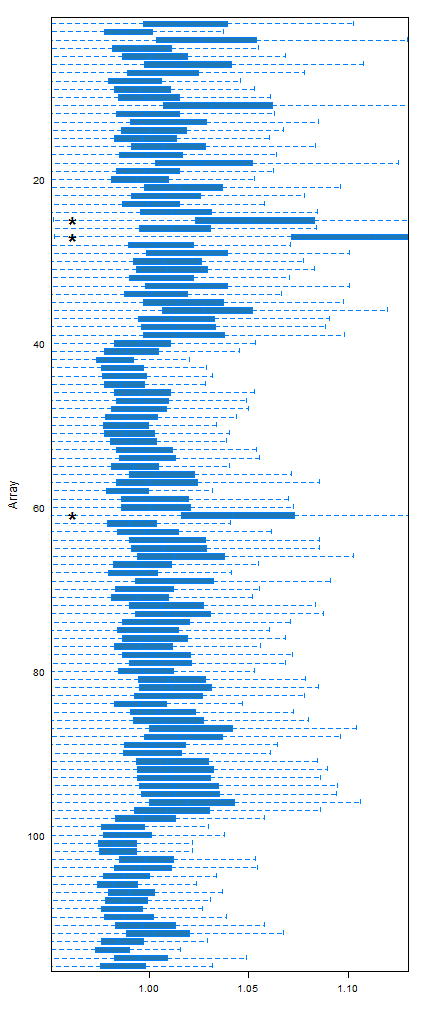

Supplement: Dataset S1 — Results of arrayQualityMetrics analysis. Only html data can be found in directories, pdf files were deleted due to size restrictions. (ZIP) [file pone.0080751.s009.zip › Supple/Gudjonsson et al/nuse.png]

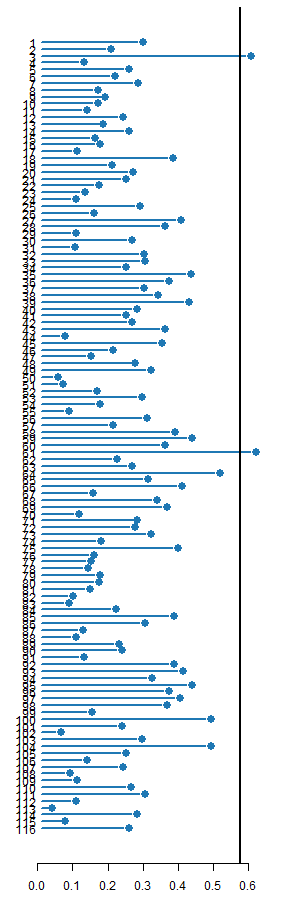

Supplement: Dataset S1 — Results of arrayQualityMetrics analysis. Only html data can be found in directories, pdf files were deleted due to size restrictions. (ZIP) [file pone.0080751.s009.zip › Supple/Gudjonsson et al/outbox.png]

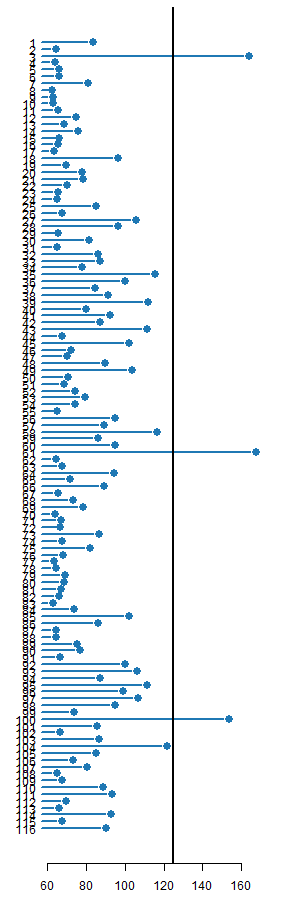

Supplement: Dataset S1 — Results of arrayQualityMetrics analysis. Only html data can be found in directories, pdf files were deleted due to size restrictions. (ZIP) [file pone.0080751.s009.zip › Supple/Gudjonsson et al/outhm.png]

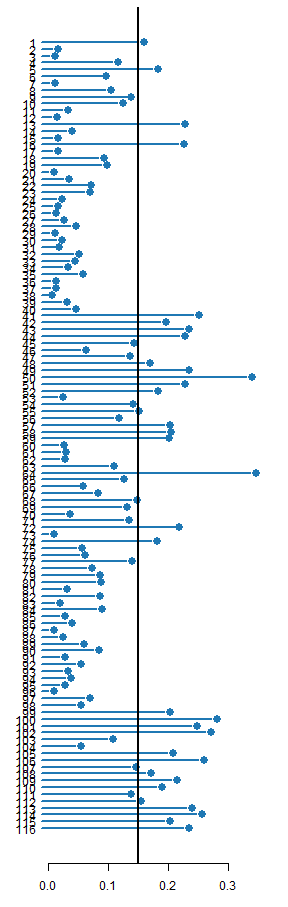

Supplement: Dataset S1 — Results of arrayQualityMetrics analysis. Only html data can be found in directories, pdf files were deleted due to size restrictions. (ZIP) [file pone.0080751.s009.zip › Supple/Gudjonsson et al/outma.png]

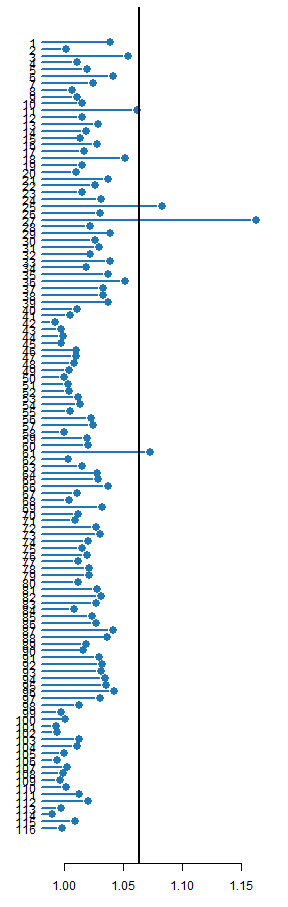

Supplement: Dataset S1 — Results of arrayQualityMetrics analysis. Only html data can be found in directories, pdf files were deleted due to size restrictions. (ZIP) [file pone.0080751.s009.zip › Supple/Gudjonsson et al/outnuse.png]

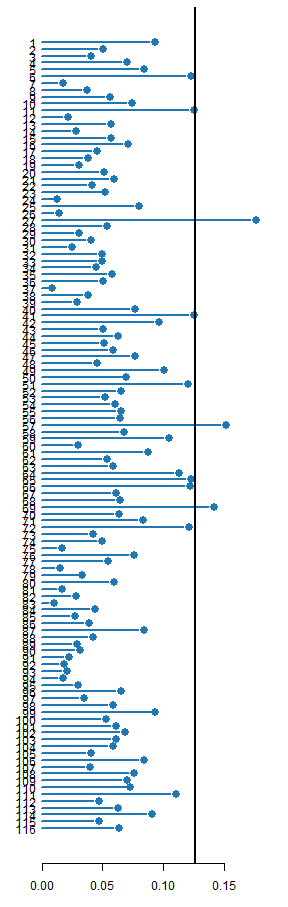

Supplement: Dataset S1 — Results of arrayQualityMetrics analysis. Only html data can be found in directories, pdf files were deleted due to size restrictions. (ZIP) [file pone.0080751.s009.zip › Supple/Gudjonsson et al/outrle.png]

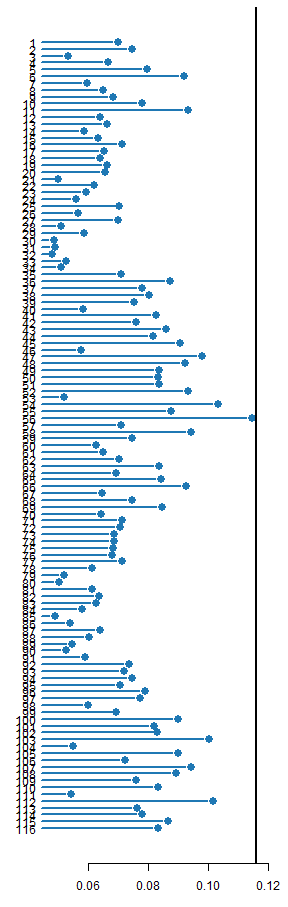

Supplement: Dataset S1 — Results of arrayQualityMetrics analysis. Only html data can be found in directories, pdf files were deleted due to size restrictions. (ZIP) [file pone.0080751.s009.zip › Supple/Gudjonsson et al/outspm.png]

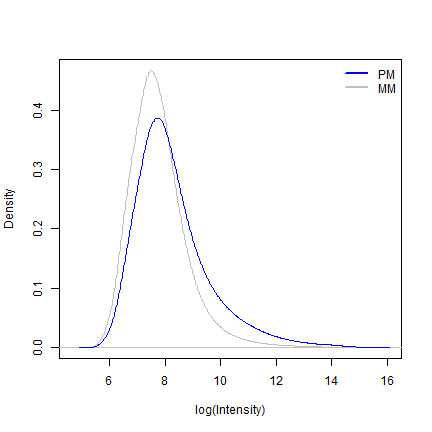

Supplement: Dataset S1 — Results of arrayQualityMetrics analysis. Only html data can be found in directories, pdf files were deleted due to size restrictions. (ZIP) [file pone.0080751.s009.zip › Supple/Gudjonsson et al/pmmm.png]

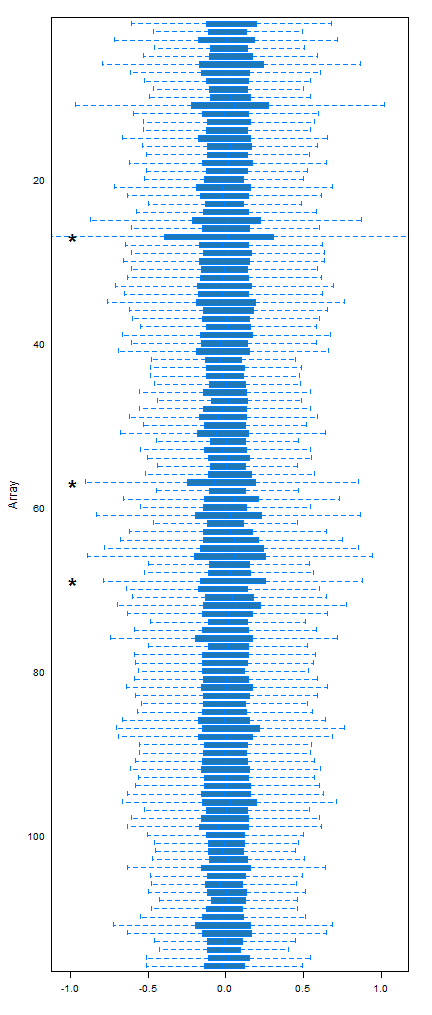

Supplement: Dataset S1 — Results of arrayQualityMetrics analysis. Only html data can be found in directories, pdf files were deleted due to size restrictions. (ZIP) [file pone.0080751.s009.zip › Supple/Gudjonsson et al/rle.png]

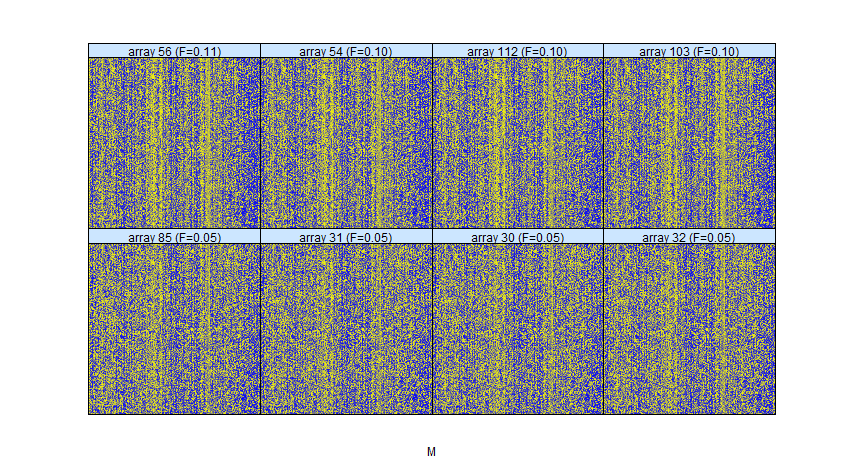

Supplement: Dataset S1 — Results of arrayQualityMetrics analysis. Only html data can be found in directories, pdf files were deleted due to size restrictions. (ZIP) [file pone.0080751.s009.zip › Supple/Gudjonsson et al/spm.png]

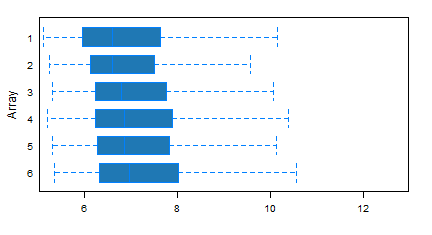

Supplement: Dataset S1 — Results of arrayQualityMetrics analysis. Only html data can be found in directories, pdf files were deleted due to size restrictions. (ZIP) [file pone.0080751.s009.zip › Supple/Johnsson-Huang et al/box.png]

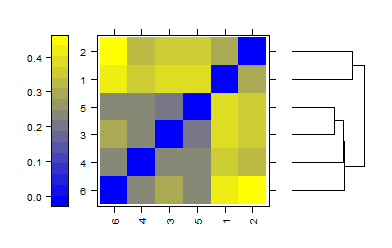

Supplement: Dataset S1 — Results of arrayQualityMetrics analysis. Only html data can be found in directories, pdf files were deleted due to size restrictions. (ZIP) [file pone.0080751.s009.zip › Supple/Johnsson-Huang et al/hm.png]

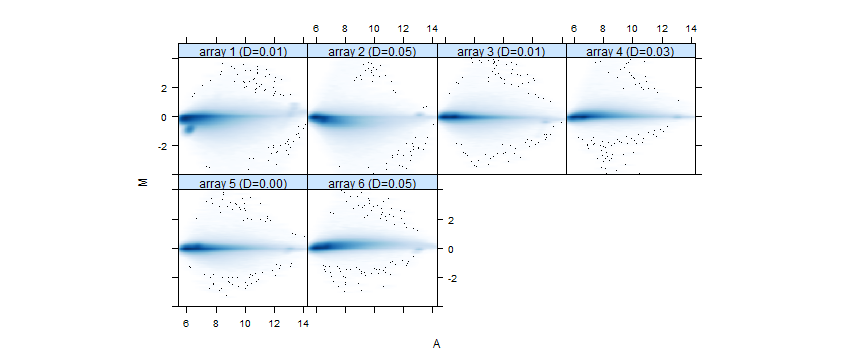

Supplement: Dataset S1 — Results of arrayQualityMetrics analysis. Only html data can be found in directories, pdf files were deleted due to size restrictions. (ZIP) [file pone.0080751.s009.zip › Supple/Johnsson-Huang et al/ma.png]

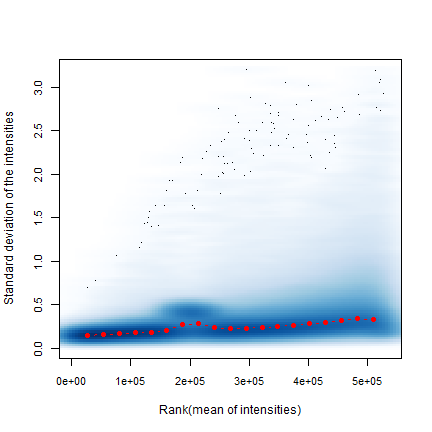

Supplement: Dataset S1 — Results of arrayQualityMetrics analysis. Only html data can be found in directories, pdf files were deleted due to size restrictions. (ZIP) [file pone.0080751.s009.zip › Supple/Johnsson-Huang et al/msd.png]

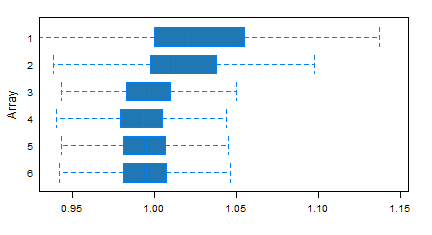

Supplement: Dataset S1 — Results of arrayQualityMetrics analysis. Only html data can be found in directories, pdf files were deleted due to size restrictions. (ZIP) [file pone.0080751.s009.zip › Supple/Johnsson-Huang et al/nuse.png]

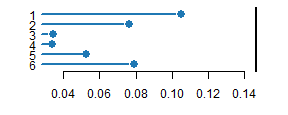

Supplement: Dataset S1 — Results of arrayQualityMetrics analysis. Only html data can be found in directories, pdf files were deleted due to size restrictions. (ZIP) [file pone.0080751.s009.zip › Supple/Johnsson-Huang et al/outbox.png]

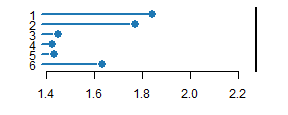

Supplement: Dataset S1 — Results of arrayQualityMetrics analysis. Only html data can be found in directories, pdf files were deleted due to size restrictions. (ZIP) [file pone.0080751.s009.zip › Supple/Johnsson-Huang et al/outhm.png]

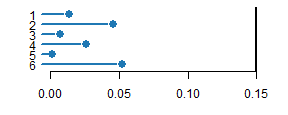

Supplement: Dataset S1 — Results of arrayQualityMetrics analysis. Only html data can be found in directories, pdf files were deleted due to size restrictions. (ZIP) [file pone.0080751.s009.zip › Supple/Johnsson-Huang et al/outma.png]

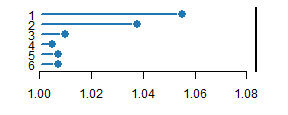

Supplement: Dataset S1 — Results of arrayQualityMetrics analysis. Only html data can be found in directories, pdf files were deleted due to size restrictions. (ZIP) [file pone.0080751.s009.zip › Supple/Johnsson-Huang et al/outnuse.png]

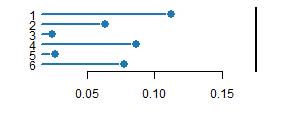

Supplement: Dataset S1 — Results of arrayQualityMetrics analysis. Only html data can be found in directories, pdf files were deleted due to size restrictions. (ZIP) [file pone.0080751.s009.zip › Supple/Johnsson-Huang et al/outrle.png]

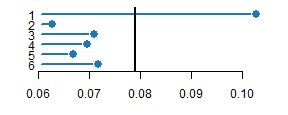

Supplement: Dataset S1 — Results of arrayQualityMetrics analysis. Only html data can be found in directories, pdf files were deleted due to size restrictions. (ZIP) [file pone.0080751.s009.zip › Supple/Johnsson-Huang et al/outspm.png]

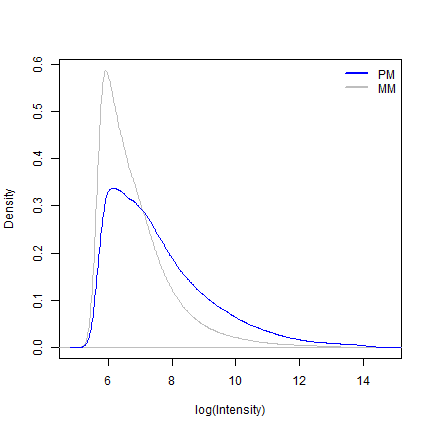

Supplement: Dataset S1 — Results of arrayQualityMetrics analysis. Only html data can be found in directories, pdf files were deleted due to size restrictions. (ZIP) [file pone.0080751.s009.zip › Supple/Johnsson-Huang et al/pmmm.png]

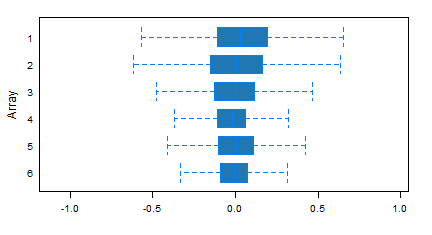

Supplement: Dataset S1 — Results of arrayQualityMetrics analysis. Only html data can be found in directories, pdf files were deleted due to size restrictions. (ZIP) [file pone.0080751.s009.zip › Supple/Johnsson-Huang et al/rle.png]

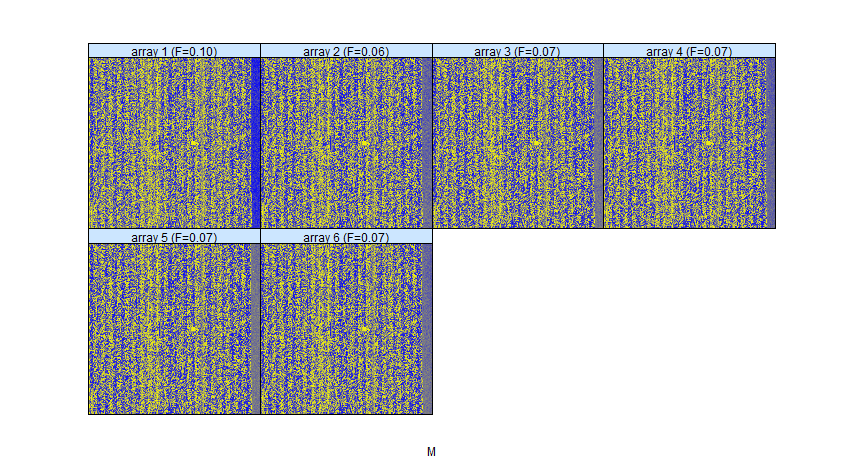

Supplement: Dataset S1 — Results of arrayQualityMetrics analysis. Only html data can be found in directories, pdf files were deleted due to size restrictions. (ZIP) [file pone.0080751.s009.zip › Supple/Johnsson-Huang et al/spm.png]

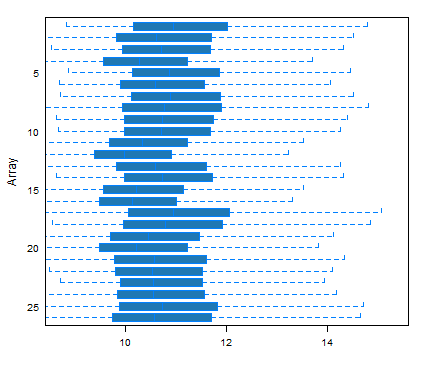

Supplement: Dataset S1 — Results of arrayQualityMetrics analysis. Only html data can be found in directories, pdf files were deleted due to size restrictions. (ZIP) [file pone.0080751.s009.zip › Supple/Reischl et al/box.png]

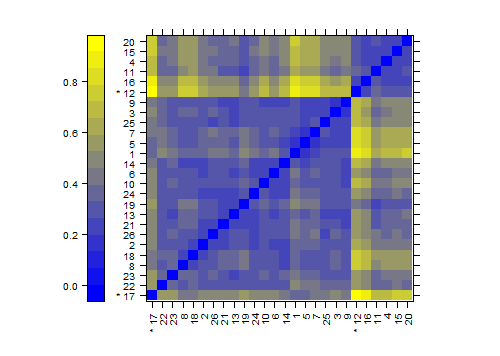

Supplement: Dataset S1 — Results of arrayQualityMetrics analysis. Only html data can be found in directories, pdf files were deleted due to size restrictions. (ZIP) [file pone.0080751.s009.zip › Supple/Reischl et al/hm.png]

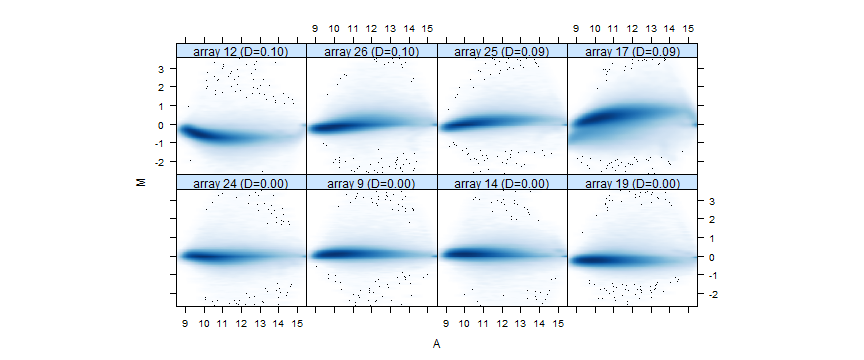

Supplement: Dataset S1 — Results of arrayQualityMetrics analysis. Only html data can be found in directories, pdf files were deleted due to size restrictions. (ZIP) [file pone.0080751.s009.zip › Supple/Reischl et al/ma.png]

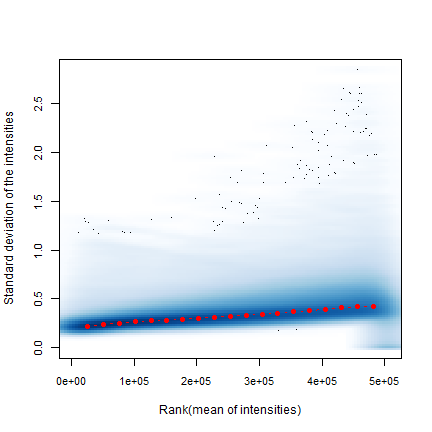

Supplement: Dataset S1 — Results of arrayQualityMetrics analysis. Only html data can be found in directories, pdf files were deleted due to size restrictions. (ZIP) [file pone.0080751.s009.zip › Supple/Reischl et al/msd.png]

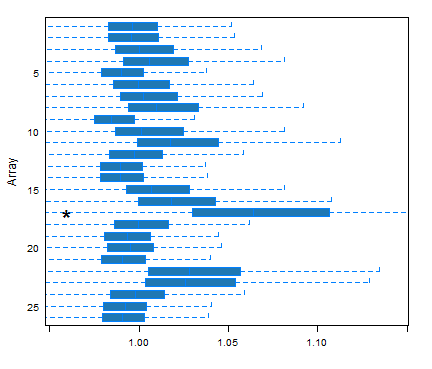

Supplement: Dataset S1 — Results of arrayQualityMetrics analysis. Only html data can be found in directories, pdf files were deleted due to size restrictions. (ZIP) [file pone.0080751.s009.zip › Supple/Reischl et al/nuse.png]

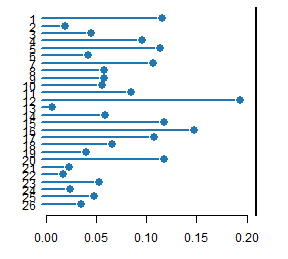

Supplement: Dataset S1 — Results of arrayQualityMetrics analysis. Only html data can be found in directories, pdf files were deleted due to size restrictions. (ZIP) [file pone.0080751.s009.zip › Supple/Reischl et al/outbox.png]

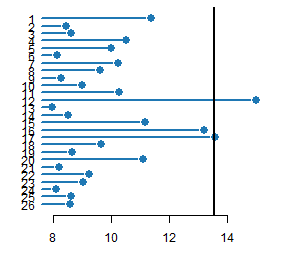

Supplement: Dataset S1 — Results of arrayQualityMetrics analysis. Only html data can be found in directories, pdf files were deleted due to size restrictions. (ZIP) [file pone.0080751.s009.zip › Supple/Reischl et al/outhm.png]

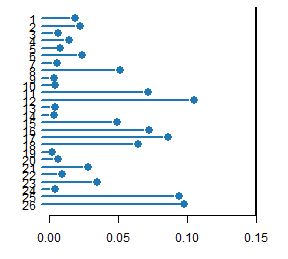

Supplement: Dataset S1 — Results of arrayQualityMetrics analysis. Only html data can be found in directories, pdf files were deleted due to size restrictions. (ZIP) [file pone.0080751.s009.zip › Supple/Reischl et al/outma.png]

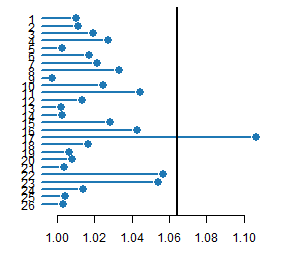

Supplement: Dataset S1 — Results of arrayQualityMetrics analysis. Only html data can be found in directories, pdf files were deleted due to size restrictions. (ZIP) [file pone.0080751.s009.zip › Supple/Reischl et al/outnuse.png]

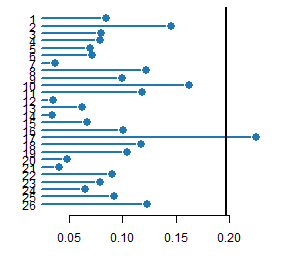

Supplement: Dataset S1 — Results of arrayQualityMetrics analysis. Only html data can be found in directories, pdf files were deleted due to size restrictions. (ZIP) [file pone.0080751.s009.zip › Supple/Reischl et al/outrle.png]

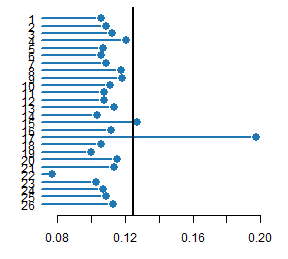

Supplement: Dataset S1 — Results of arrayQualityMetrics analysis. Only html data can be found in directories, pdf files were deleted due to size restrictions. (ZIP) [file pone.0080751.s009.zip › Supple/Reischl et al/outspm.png]

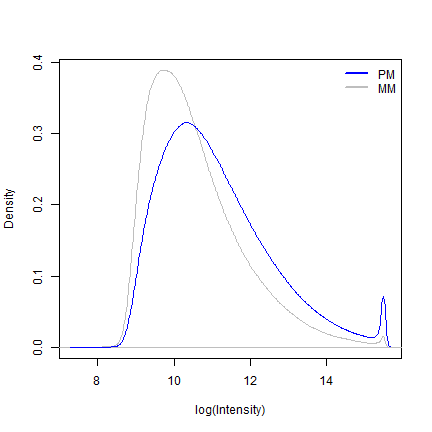

Supplement: Dataset S1 — Results of arrayQualityMetrics analysis. Only html data can be found in directories, pdf files were deleted due to size restrictions. (ZIP) [file pone.0080751.s009.zip › Supple/Reischl et al/pmmm.png]

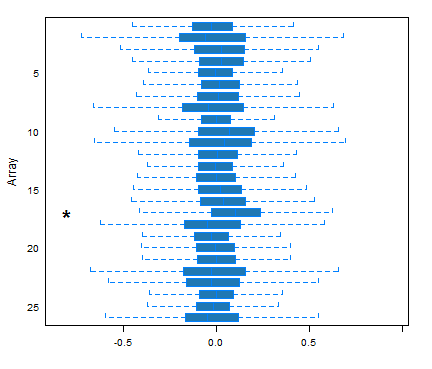

Supplement: Dataset S1 — Results of arrayQualityMetrics analysis. Only html data can be found in directories, pdf files were deleted due to size restrictions. (ZIP) [file pone.0080751.s009.zip › Supple/Reischl et al/rle.png]

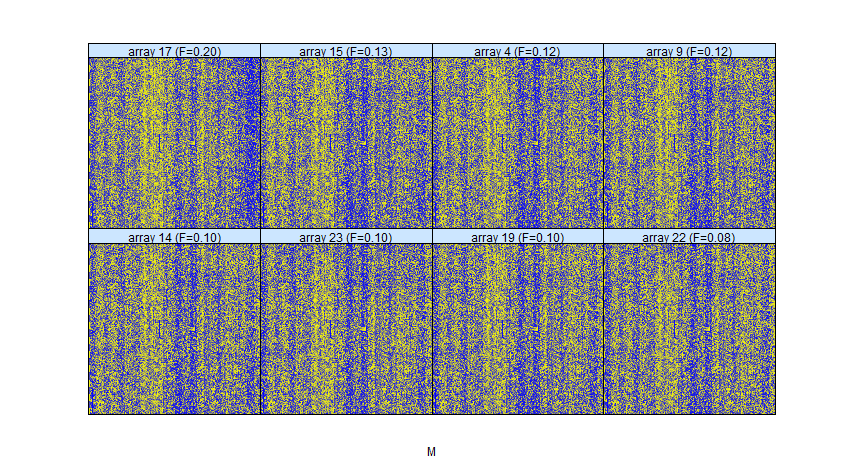

Supplement: Dataset S1 — Results of arrayQualityMetrics analysis. Only html data can be found in directories, pdf files were deleted due to size restrictions. (ZIP) [file pone.0080751.s009.zip › Supple/Reischl et al/spm.png]

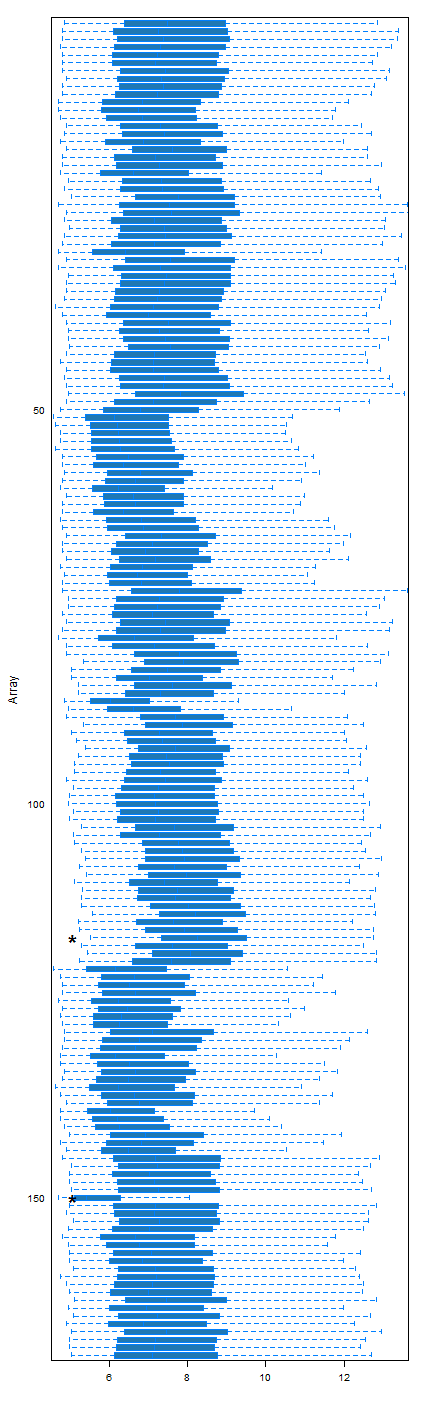

Supplement: Dataset S1 — Results of arrayQualityMetrics analysis. Only html data can be found in directories, pdf files were deleted due to size restrictions. (ZIP) [file pone.0080751.s009.zip › Supple/Suarez-Farinas et al/box.png]

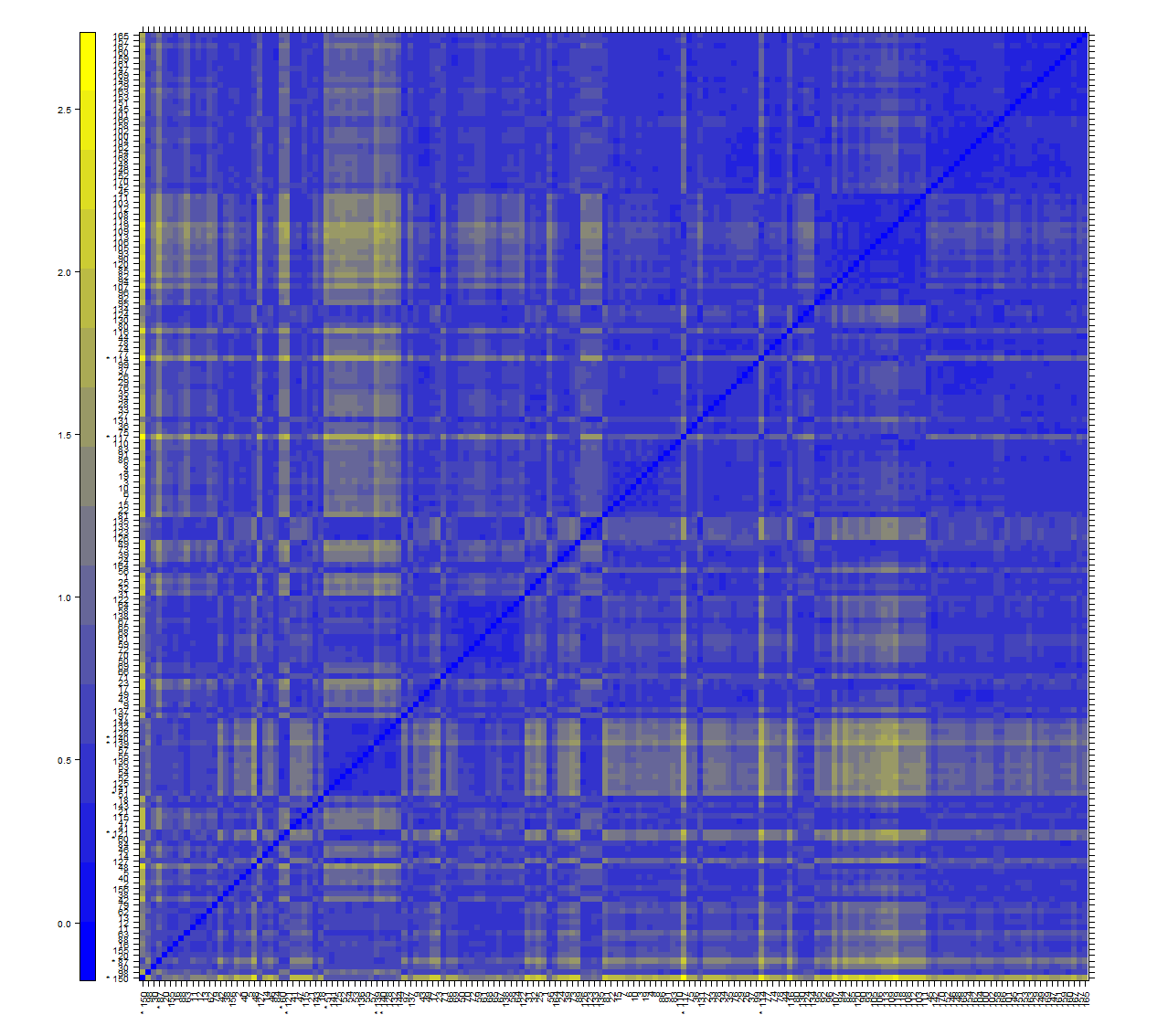

Supplement: Dataset S1 — Results of arrayQualityMetrics analysis. Only html data can be found in directories, pdf files were deleted due to size restrictions. (ZIP) [file pone.0080751.s009.zip › Supple/Suarez-Farinas et al/hm.png]

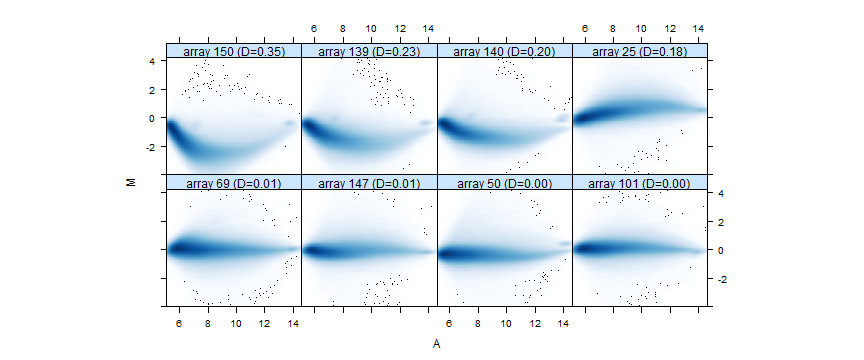

Supplement: Dataset S1 — Results of arrayQualityMetrics analysis. Only html data can be found in directories, pdf files were deleted due to size restrictions. (ZIP) [file pone.0080751.s009.zip › Supple/Suarez-Farinas et al/ma.png]

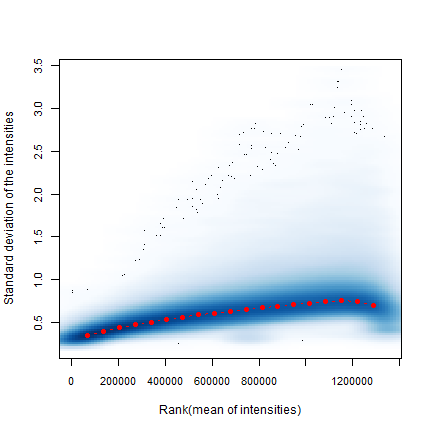

Supplement: Dataset S1 — Results of arrayQualityMetrics analysis. Only html data can be found in directories, pdf files were deleted due to size restrictions. (ZIP) [file pone.0080751.s009.zip › Supple/Suarez-Farinas et al/msd.png]

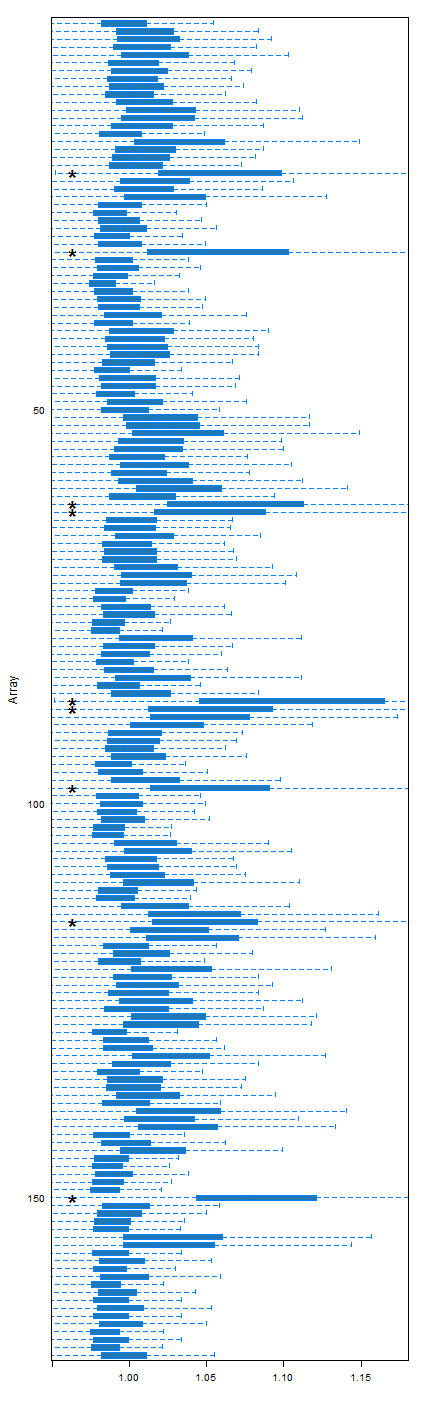

Supplement: Dataset S1 — Results of arrayQualityMetrics analysis. Only html data can be found in directories, pdf files were deleted due to size restrictions. (ZIP) [file pone.0080751.s009.zip › Supple/Suarez-Farinas et al/nuse.png]

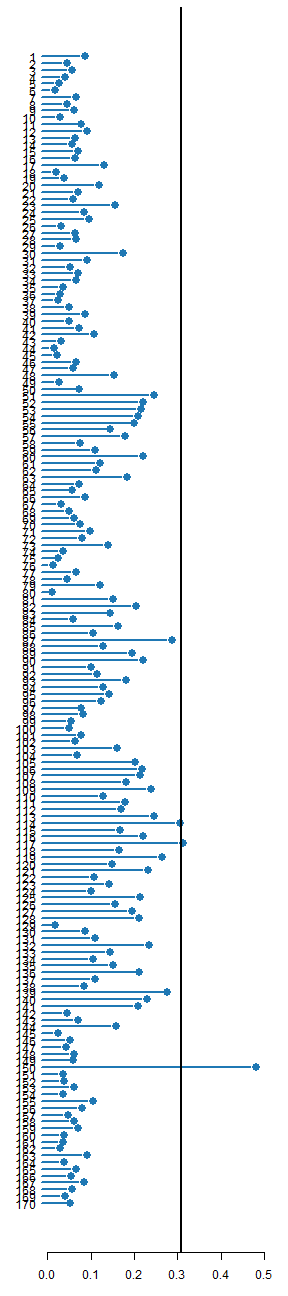

Supplement: Dataset S1 — Results of arrayQualityMetrics analysis. Only html data can be found in directories, pdf files were deleted due to size restrictions. (ZIP) [file pone.0080751.s009.zip › Supple/Suarez-Farinas et al/outbox.png]

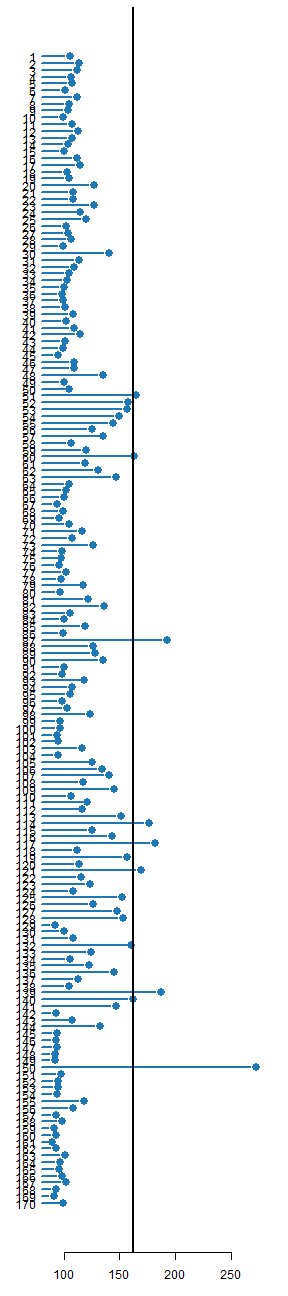

Supplement: Dataset S1 — Results of arrayQualityMetrics analysis. Only html data can be found in directories, pdf files were deleted due to size restrictions. (ZIP) [file pone.0080751.s009.zip › Supple/Suarez-Farinas et al/outhm.png]

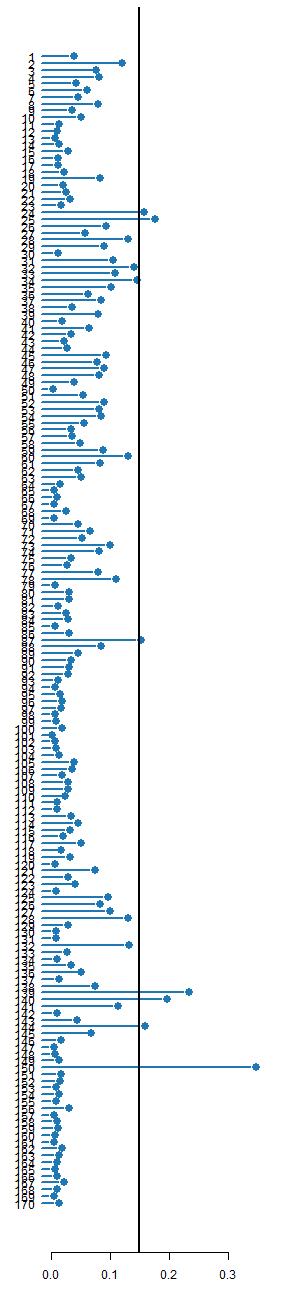

Supplement: Dataset S1 — Results of arrayQualityMetrics analysis. Only html data can be found in directories, pdf files were deleted due to size restrictions. (ZIP) [file pone.0080751.s009.zip › Supple/Suarez-Farinas et al/outma.png]

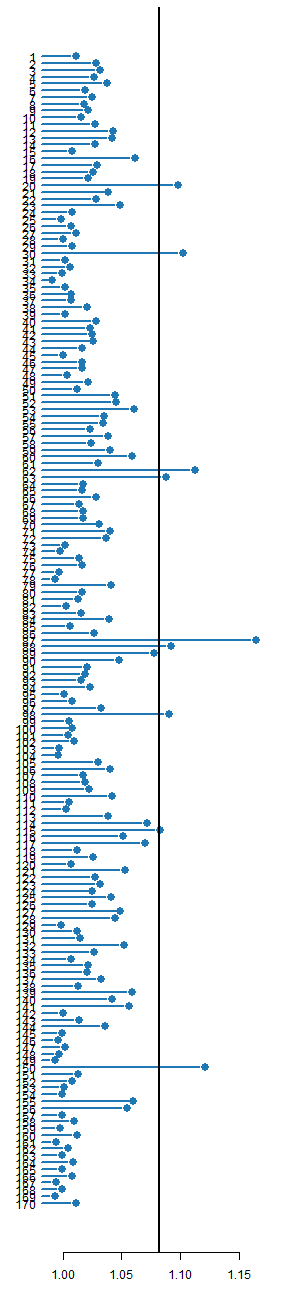

Supplement: Dataset S1 — Results of arrayQualityMetrics analysis. Only html data can be found in directories, pdf files were deleted due to size restrictions. (ZIP) [file pone.0080751.s009.zip › Supple/Suarez-Farinas et al/outnuse.png]

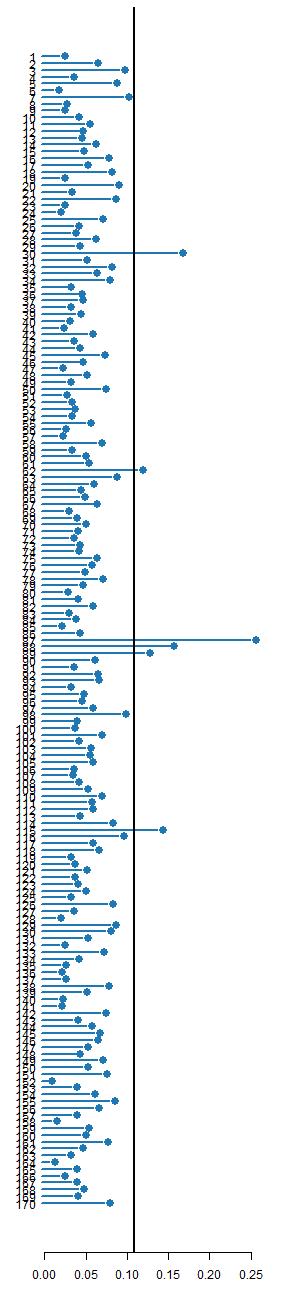

Supplement: Dataset S1 — Results of arrayQualityMetrics analysis. Only html data can be found in directories, pdf files were deleted due to size restrictions. (ZIP) [file pone.0080751.s009.zip › Supple/Suarez-Farinas et al/outrle.png]

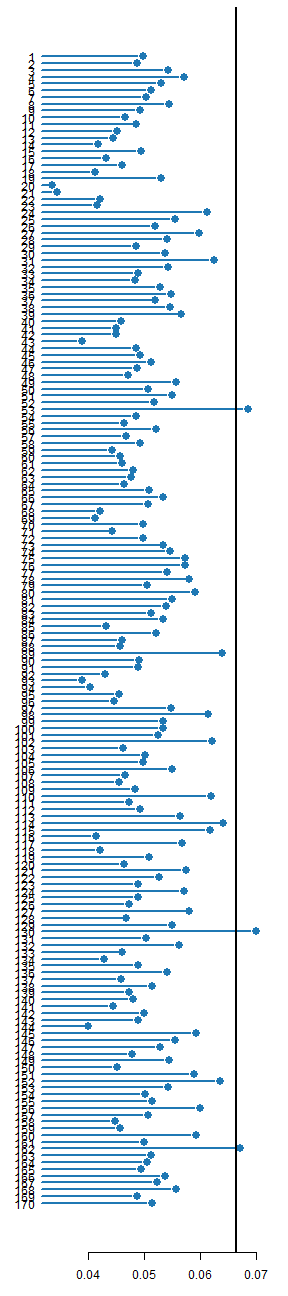

Supplement: Dataset S1 — Results of arrayQualityMetrics analysis. Only html data can be found in directories, pdf files were deleted due to size restrictions. (ZIP) [file pone.0080751.s009.zip › Supple/Suarez-Farinas et al/outspm.png]

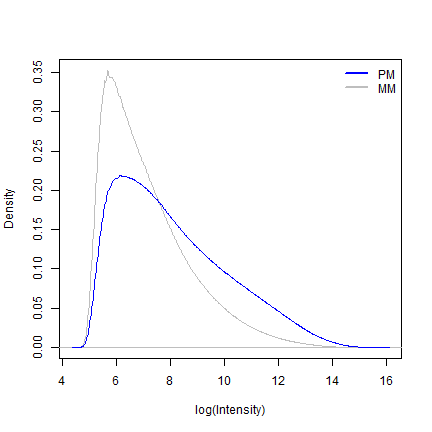

Supplement: Dataset S1 — Results of arrayQualityMetrics analysis. Only html data can be found in directories, pdf files were deleted due to size restrictions. (ZIP) [file pone.0080751.s009.zip › Supple/Suarez-Farinas et al/pmmm.png]

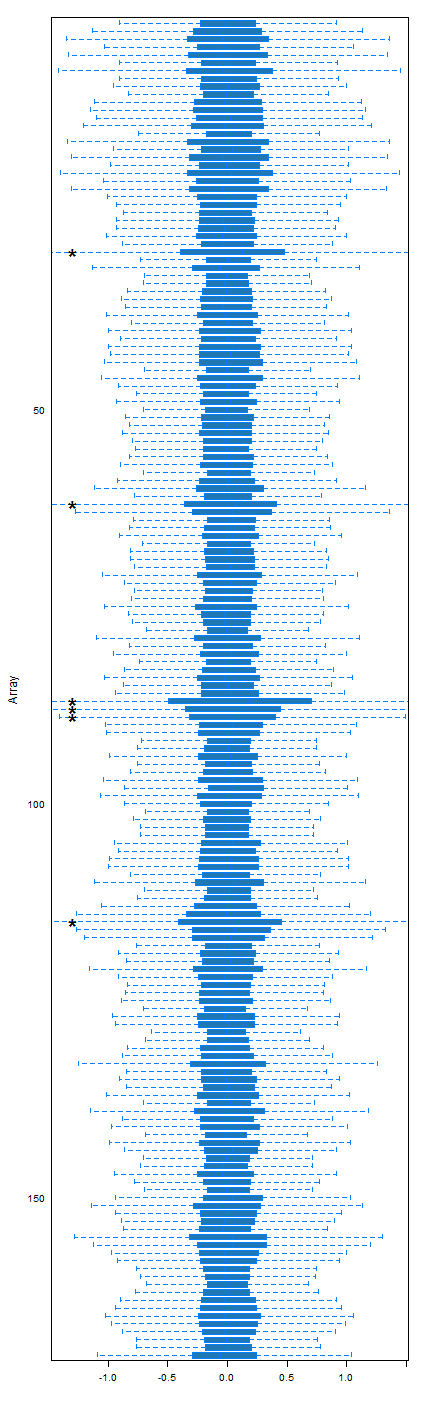

Supplement: Dataset S1 — Results of arrayQualityMetrics analysis. Only html data can be found in directories, pdf files were deleted due to size restrictions. (ZIP) [file pone.0080751.s009.zip › Supple/Suarez-Farinas et al/rle.png]

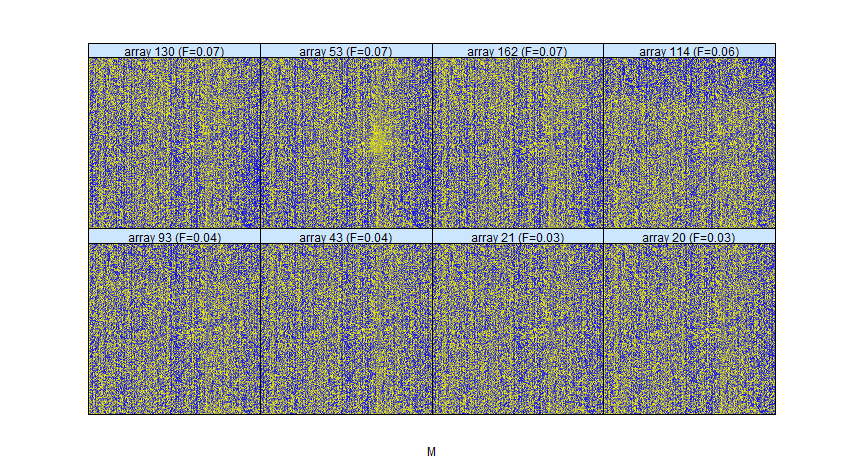

Supplement: Dataset S1 — Results of arrayQualityMetrics analysis. Only html data can be found in directories, pdf files were deleted due to size restrictions. (ZIP) [file pone.0080751.s009.zip › Supple/Suarez-Farinas et al/spm.png]

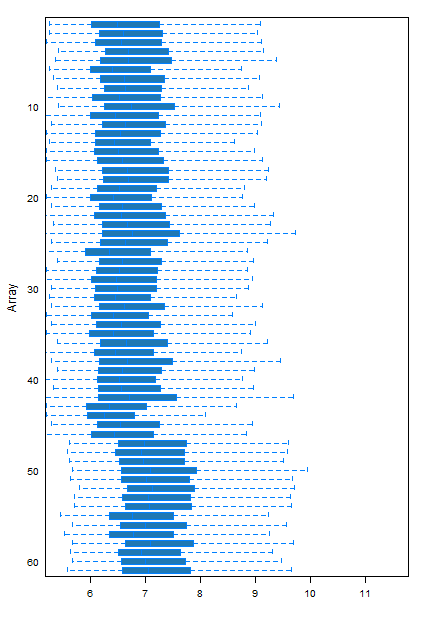

Supplement: Dataset S1 — Results of arrayQualityMetrics analysis. Only html data can be found in directories, pdf files were deleted due to size restrictions. (ZIP) [file pone.0080751.s009.zip › Supple/Yao et al/box.png]

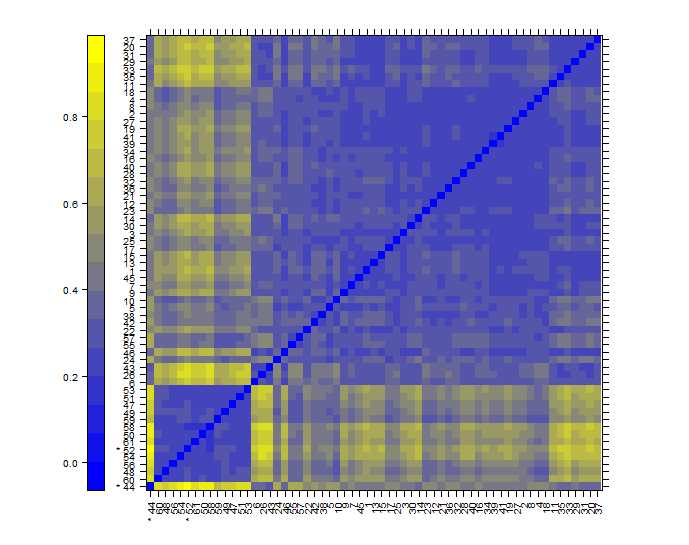

Supplement: Dataset S1 — Results of arrayQualityMetrics analysis. Only html data can be found in directories, pdf files were deleted due to size restrictions. (ZIP) [file pone.0080751.s009.zip › Supple/Yao et al/hm.png]

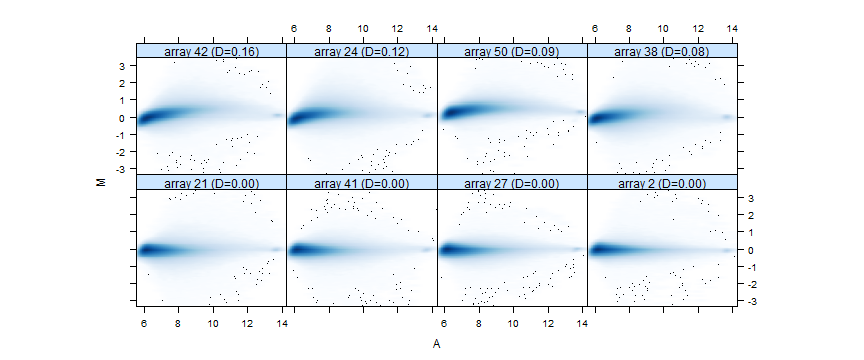

Supplement: Dataset S1 — Results of arrayQualityMetrics analysis. Only html data can be found in directories, pdf files were deleted due to size restrictions. (ZIP) [file pone.0080751.s009.zip › Supple/Yao et al/ma.png]

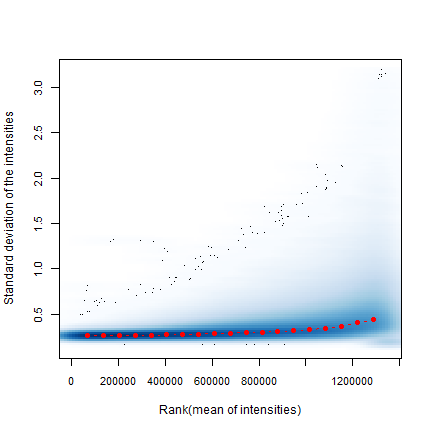

Supplement: Dataset S1 — Results of arrayQualityMetrics analysis. Only html data can be found in directories, pdf files were deleted due to size restrictions. (ZIP) [file pone.0080751.s009.zip › Supple/Yao et al/msd.png]

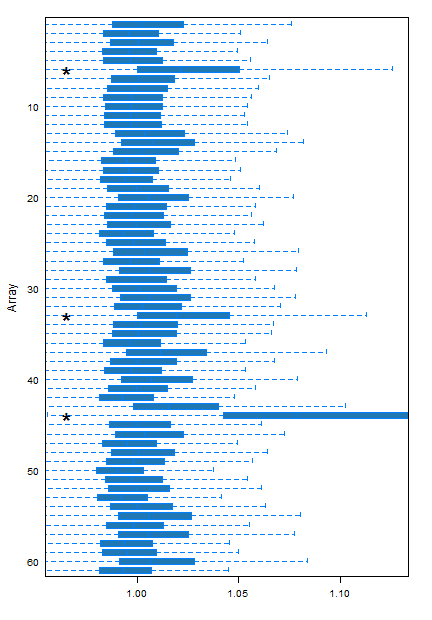

Supplement: Dataset S1 — Results of arrayQualityMetrics analysis. Only html data can be found in directories, pdf files were deleted due to size restrictions. (ZIP) [file pone.0080751.s009.zip › Supple/Yao et al/nuse.png]

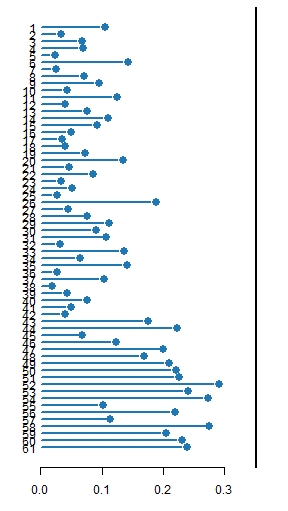

Supplement: Dataset S1 — Results of arrayQualityMetrics analysis. Only html data can be found in directories, pdf files were deleted due to size restrictions. (ZIP) [file pone.0080751.s009.zip › Supple/Yao et al/outbox.png]

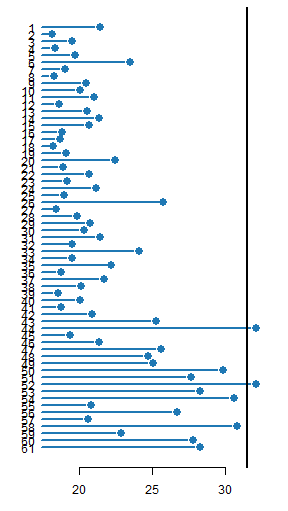

Supplement: Dataset S1 — Results of arrayQualityMetrics analysis. Only html data can be found in directories, pdf files were deleted due to size restrictions. (ZIP) [file pone.0080751.s009.zip › Supple/Yao et al/outhm.png]

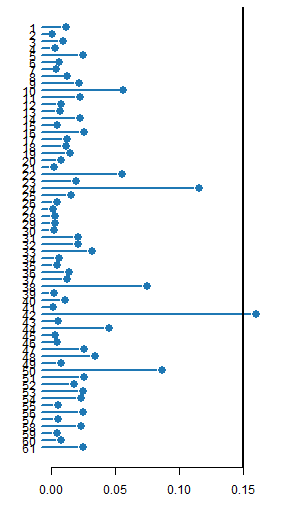

Supplement: Dataset S1 — Results of arrayQualityMetrics analysis. Only html data can be found in directories, pdf files were deleted due to size restrictions. (ZIP) [file pone.0080751.s009.zip › Supple/Yao et al/outma.png]

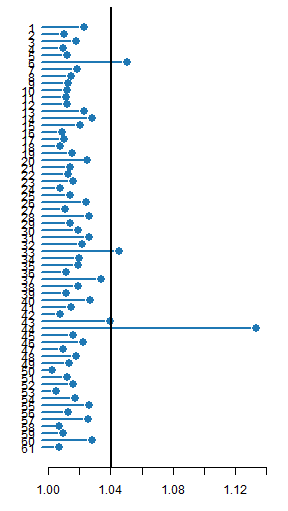

Supplement: Dataset S1 — Results of arrayQualityMetrics analysis. Only html data can be found in directories, pdf files were deleted due to size restrictions. (ZIP) [file pone.0080751.s009.zip › Supple/Yao et al/outnuse.png]

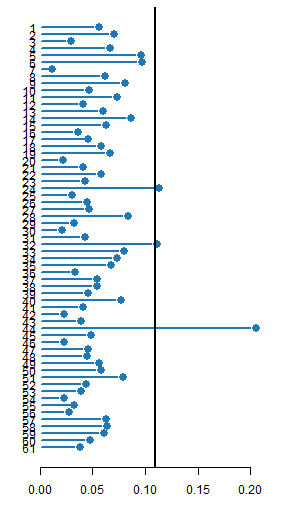

Supplement: Dataset S1 — Results of arrayQualityMetrics analysis. Only html data can be found in directories, pdf files were deleted due to size restrictions. (ZIP) [file pone.0080751.s009.zip › Supple/Yao et al/outrle.png]

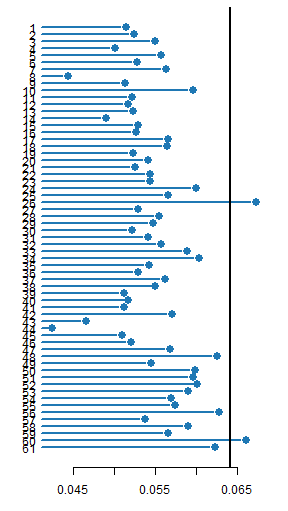

Supplement: Dataset S1 — Results of arrayQualityMetrics analysis. Only html data can be found in directories, pdf files were deleted due to size restrictions. (ZIP) [file pone.0080751.s009.zip › Supple/Yao et al/outspm.png]

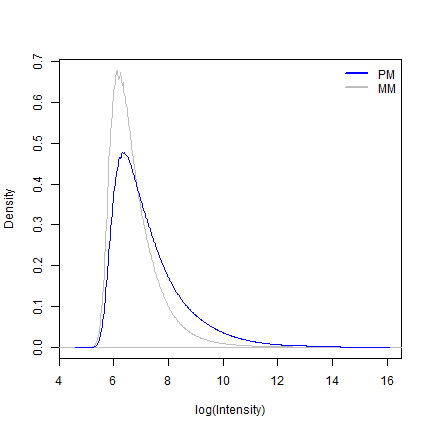

Supplement: Dataset S1 — Results of arrayQualityMetrics analysis. Only html data can be found in directories, pdf files were deleted due to size restrictions. (ZIP) [file pone.0080751.s009.zip › Supple/Yao et al/pmmm.png]

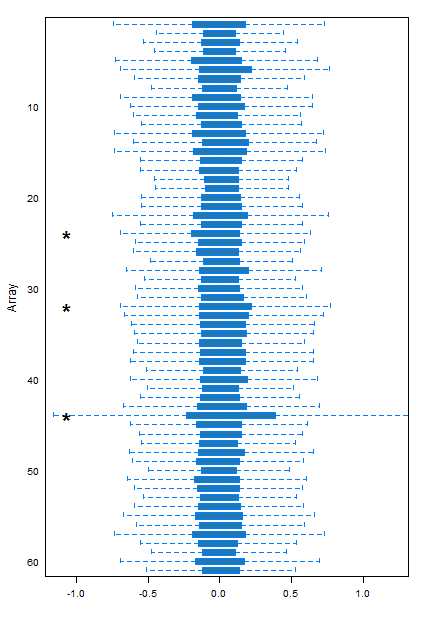

Supplement: Dataset S1 — Results of arrayQualityMetrics analysis. Only html data can be found in directories, pdf files were deleted due to size restrictions. (ZIP) [file pone.0080751.s009.zip › Supple/Yao et al/rle.png]

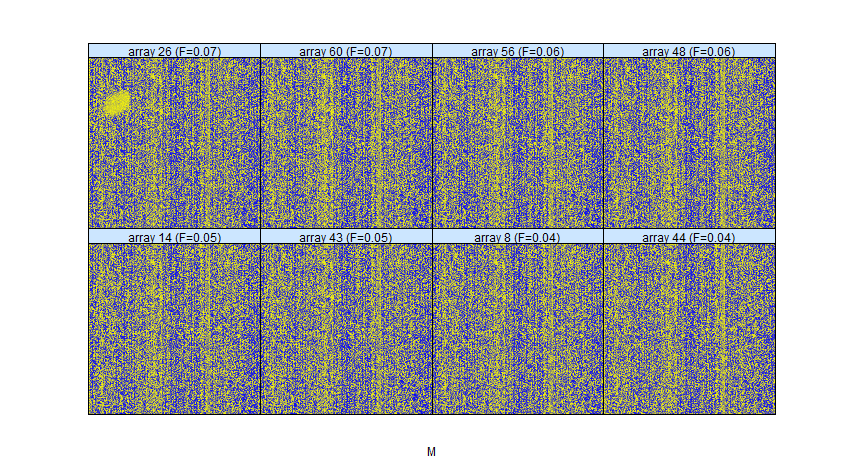

Supplement: Dataset S1 — Results of arrayQualityMetrics analysis. Only html data can be found in directories, pdf files were deleted due to size restrictions. (ZIP) [file pone.0080751.s009.zip › Supple/Yao et al/spm.png]

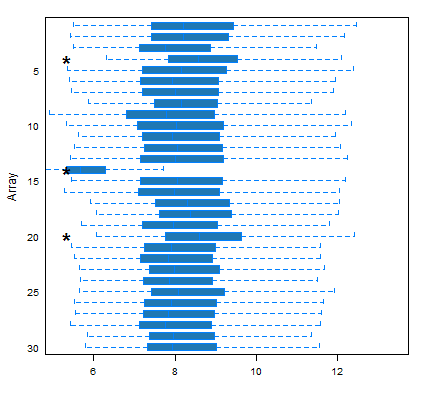

Supplement: Dataset S1 — Results of arrayQualityMetrics analysis. Only html data can be found in directories, pdf files were deleted due to size restrictions. (ZIP) [file pone.0080751.s009.zip › Supple/Zaba et al/box.png]

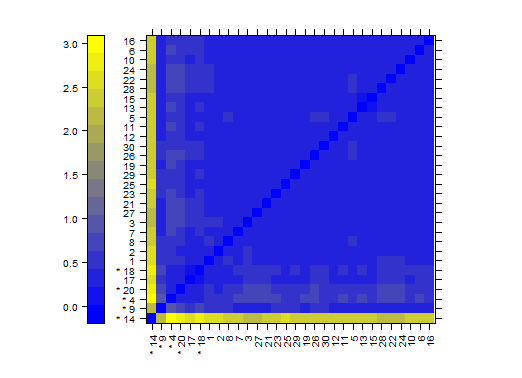

Supplement: Dataset S1 — Results of arrayQualityMetrics analysis. Only html data can be found in directories, pdf files were deleted due to size restrictions. (ZIP) [file pone.0080751.s009.zip › Supple/Zaba et al/hm.png]

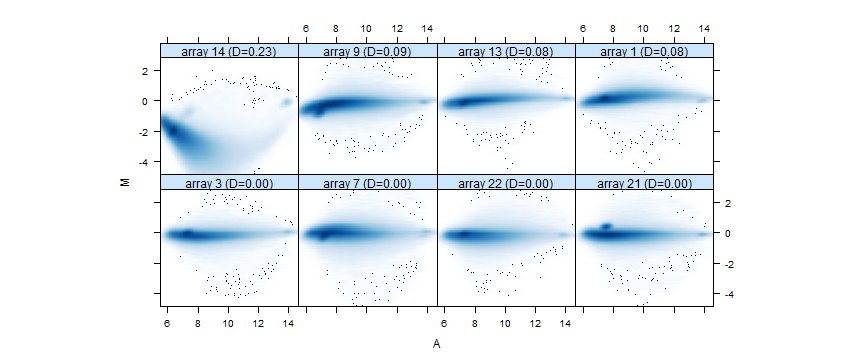

Supplement: Dataset S1 — Results of arrayQualityMetrics analysis. Only html data can be found in directories, pdf files were deleted due to size restrictions. (ZIP) [file pone.0080751.s009.zip › Supple/Zaba et al/ma.png]

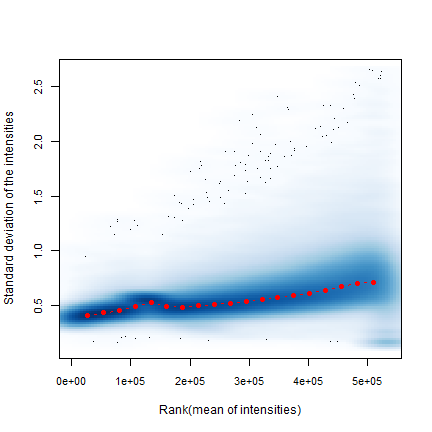

Supplement: Dataset S1 — Results of arrayQualityMetrics analysis. Only html data can be found in directories, pdf files were deleted due to size restrictions. (ZIP) [file pone.0080751.s009.zip › Supple/Zaba et al/msd.png]

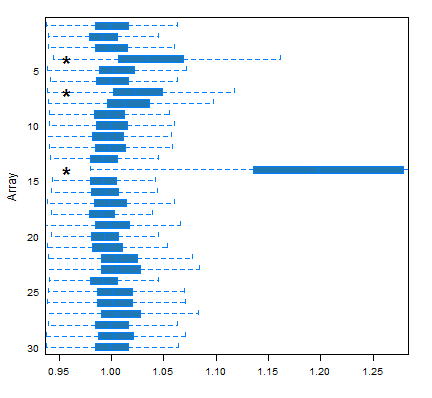

Supplement: Dataset S1 — Results of arrayQualityMetrics analysis. Only html data can be found in directories, pdf files were deleted due to size restrictions. (ZIP) [file pone.0080751.s009.zip › Supple/Zaba et al/nuse.png]

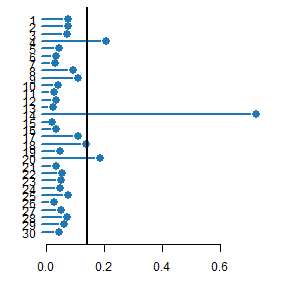

Supplement: Dataset S1 — Results of arrayQualityMetrics analysis. Only html data can be found in directories, pdf files were deleted due to size restrictions. (ZIP) [file pone.0080751.s009.zip › Supple/Zaba et al/outbox.png]

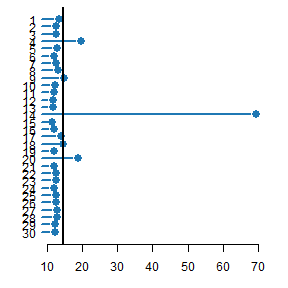

Supplement: Dataset S1 — Results of arrayQualityMetrics analysis. Only html data can be found in directories, pdf files were deleted due to size restrictions. (ZIP) [file pone.0080751.s009.zip › Supple/Zaba et al/outhm.png]

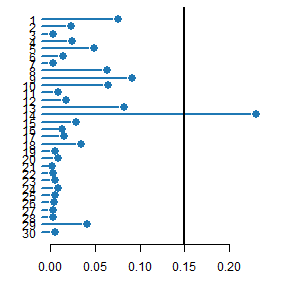

Supplement: Dataset S1 — Results of arrayQualityMetrics analysis. Only html data can be found in directories, pdf files were deleted due to size restrictions. (ZIP) [file pone.0080751.s009.zip › Supple/Zaba et al/outma.png]

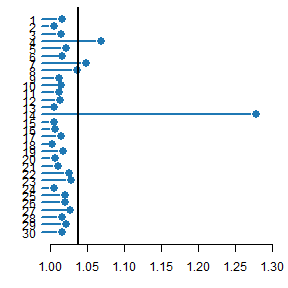

Supplement: Dataset S1 — Results of arrayQualityMetrics analysis. Only html data can be found in directories, pdf files were deleted due to size restrictions. (ZIP) [file pone.0080751.s009.zip › Supple/Zaba et al/outnuse.png]

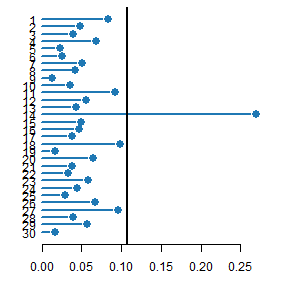

Supplement: Dataset S1 — Results of arrayQualityMetrics analysis. Only html data can be found in directories, pdf files were deleted due to size restrictions. (ZIP) [file pone.0080751.s009.zip › Supple/Zaba et al/outrle.png]

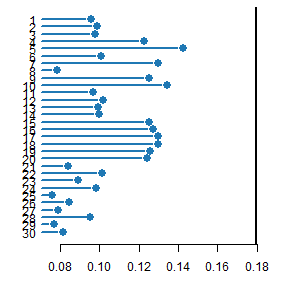

Supplement: Dataset S1 — Results of arrayQualityMetrics analysis. Only html data can be found in directories, pdf files were deleted due to size restrictions. (ZIP) [file pone.0080751.s009.zip › Supple/Zaba et al/outspm.png]

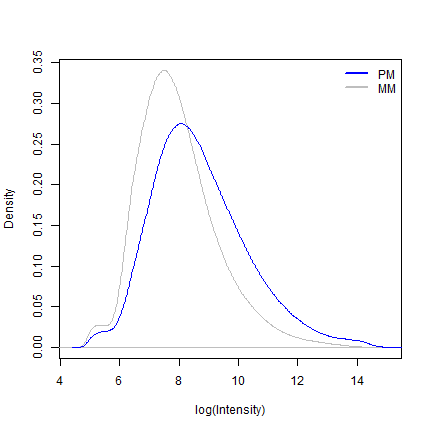

Supplement: Dataset S1 — Results of arrayQualityMetrics analysis. Only html data can be found in directories, pdf files were deleted due to size restrictions. (ZIP) [file pone.0080751.s009.zip › Supple/Zaba et al/pmmm.png]

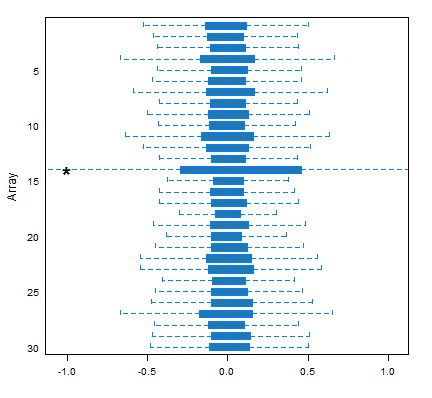

Supplement: Dataset S1 — Results of arrayQualityMetrics analysis. Only html data can be found in directories, pdf files were deleted due to size restrictions. (ZIP) [file pone.0080751.s009.zip › Supple/Zaba et al/rle.png]

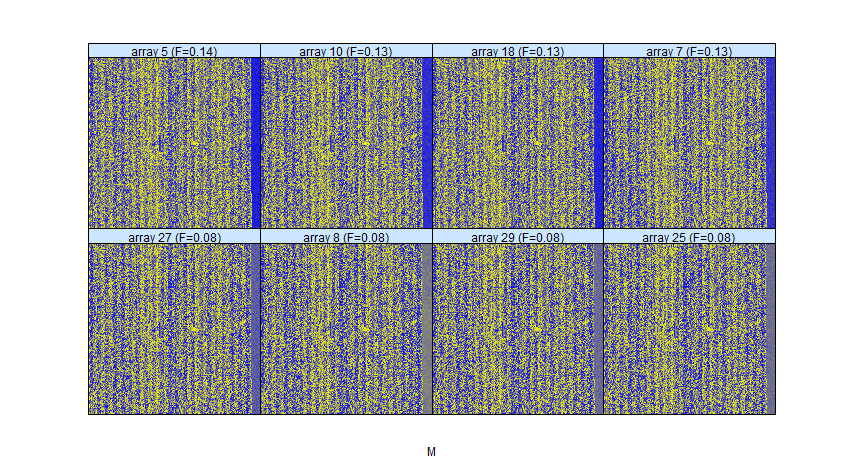

Supplement: Dataset S1 — Results of arrayQualityMetrics analysis. Only html data can be found in directories, pdf files were deleted due to size restrictions. (ZIP) [file pone.0080751.s009.zip › Supple/Zaba et al/spm.png]
