# Supplementary material for: Novel Factors in the Pathogenesis of Psoriasis and Potential Drug Candidates Are Found with Systems Biology Approach
Source: PLoS One. 2013 Nov 26;8(11):e80751. doi: 10.1371/journal.pone.0080751 (PMC3841158; doi:10.1371/journal.pone.0080751)
Supplement: Dataset S1 — Results of arrayQualityMetrics analysis. Only html data can be found in directories, pdf files were deleted due to size restrictions. (ZIP) [file pone.0080751.s009.zip › Supple/Zaba et al/index.html]

arrayQualityMetrics report for suarez3


---

# arrayQualityMetrics report for suarez3

---

  
- Section 1: Between array comparison

- Distances between arrays
- Principal Component Analysis

  
- Section 2: Array intensity distributions

- Boxplots
- Density plots
  
- Section 3: Variance mean dependence

- Standard deviation versus rank of the mean
  
- Section 4: Affymetrix specific plots

- Relative Log Expression (RLE)
- Normalized Unscaled Standard Error (NUSE)
- RNA digestion plot
- Perfect matches and mismatches
  
- Section 5: Individual array quality

- MA plots
- Spatial distribution of M

---

### Browser compatibility

This report uses recent features of HTML 5. Functionality has been tested on these browsers: Firefox 10, Chrome 17, Safari 5.1.2

---

+ Array metadata and outlier detection overview  

|  |  |  |  |  |  |  |  |  |  |  |
| --- | --- | --- | --- | --- | --- | --- | --- | --- | --- | --- |
|  | array | sampleNames | \*1 | \*2 | \*3 | \*4 | \*5 | \*6 | sample | ScanDate |
|  | 1 | GSM300749.CEL |  |  |  |  |  |  | 1 | 01/23/07 13:03:47 |
|  | 2 | GSM300750.CEL |  |  |  |  |  |  | 2 | 01/23/07 13:10:11 |
|  | 3 | GSM300755.CEL |  |  |  |  |  |  | 3 | 01/23/07 13:42:17 |
|  | 4 | GSM300756.CEL | x | x |  | x |  |  | 4 | 01/23/07 13:48:43 |
|  | 5 | GSM300761.CEL |  |  |  |  |  |  | 5 | 01/23/07 15:18:20 |
|  | 6 | GSM300762.CEL |  |  |  |  |  |  | 6 | 01/23/07 15:24:43 |
|  | 7 | GSM300767.CEL |  |  |  | x |  |  | 7 | 01/23/07 16:28:26 |
|  | 8 | GSM300768.CEL |  |  |  |  |  |  | 8 | 01/23/07 16:35:02 |
|  | 9 | GSM300773.CEL | x |  |  |  |  |  | 9 | 01/24/07 12:12:25 |
|  | 10 | GSM300774.CEL |  |  |  |  |  |  | 10 | 01/24/07 12:18:55 |
|  | 11 | GSM300779.CEL |  |  |  |  |  |  | 11 | 01/24/07 12:50:51 |
|  | 12 | GSM300780.CEL |  |  |  |  |  |  | 12 | 01/24/07 12:56:59 |
|  | 13 | GSM300785.CEL |  |  |  |  |  |  | 13 | 01/24/07 17:15:20 |
|  | 14 | GSM300786.CEL | x | x | x | x | x |  | 14 | 01/24/07 14:42:43 |
|  | 15 | GSM300791.CEL |  |  |  |  |  |  | 15 | 01/24/07 16:03:54 |
|  | 16 | GSM300792.CEL |  |  |  |  |  |  | 16 | 01/24/07 16:10:22 |
|  | 17 | GSM300797.CEL |  |  |  |  |  |  | 17 | 01/25/07 12:24:34 |
|  | 18 | GSM300798.CEL | x |  |  |  |  |  | 18 | 01/25/07 12:30:56 |
|  | 19 | GSM300803.CEL |  |  |  |  |  |  | 19 | 01/25/07 13:03:09 |
|  | 20 | GSM300804.CEL | x | x |  |  |  |  | 20 | 01/25/07 13:37:28 |
|  | 21 | GSM300809.CEL |  |  |  |  |  |  | 21 | 01/25/07 14:09:52 |
|  | 22 | GSM300810.CEL |  |  |  |  |  |  | 22 | 01/25/07 14:16:14 |
|  | 23 | GSM300814.CEL |  |  |  |  |  |  | 23 | 01/25/07 15:24:12 |
|  | 24 | GSM300815.CEL |  |  |  |  |  |  | 24 | 01/25/07 15:30:40 |
|  | 25 | GSM300820.CEL |  |  |  |  |  |  | 25 | 01/30/07 11:37:33 |
|  | 26 | GSM300821.CEL |  |  |  |  |  |  | 26 | 01/30/07 11:43:51 |
|  | 27 | GSM300826.CEL |  |  |  |  |  |  | 27 | 01/30/07 12:16:09 |
|  | 28 | GSM300827.CEL |  |  |  |  |  |  | 28 | 01/30/07 12:22:31 |
|  | 29 | GSM300832.CEL |  |  |  |  |  |  | 29 | 01/30/07 13:36:30 |
|  | 30 | GSM300833.CEL |  |  |  |  |  |  | 30 | 01/30/07 13:42:44 |

  
The columns named \*1, \*2, ... indicate the calls from the different outlier detection methods:

1. outlier detection by Distances between arrays
2. outlier detection by Boxplots
3. outlier detection by Relative Log Expression (RLE)
4. outlier detection by Normalized Unscaled Standard Error (NUSE)
5. outlier detection by MA plots
6. outlier detection by Spatial distribution of M

The outlier detection criteria are explained below in the respective sections. Arrays that were called outliers by at least one criterion are marked by checkbox selection in this table, and are indicated by highlighted lines or points in some of the plots below. By clicking the checkboxes in the table, or on the corresponding points/lines in the plots, you can modify the selection. To reset the selection, reload the HTML page in your browser.  
  
At the scope covered by this software, outlier detection is a poorly defined question, and there is no 'right' or 'wrong' answer. These are hints which are intended to be followed up manually. If you want to automate outlier detection, you need to limit the scope to a particular platform and experimental design, and then choose and calibrate the metrics used.

---

## Section 1: Between array comparison

- Figure 1: Distances between arrays.  

**Figure 1** (PDF file) shows a false color heatmap of the distances between arrays. The color scale is chosen to cover the range of distances encountered in the dataset. Patterns in this plot can indicate clustering of the arrays either because of intended biological or unintended experimental factors (batch effects). The distance *dab* between two arrays *a* and *b* is computed as the mean absolute difference (L1-distance) between the data of the arrays (using the data from all probes without filtering). In formula, *dab* = mean | *Mai - Mbi* |, where *Mai* is the value of the *i*-th probe on the *a*-th array. Outlier detection was performed by looking for arrays for which the sum of the distances to all other arrays, *Sa* = Σ*b* *dab* was exceptionally large. 5 such arrays were detected, and they are marked by an asterisk, \*.

+ Figure 2: Outlier detection for Distances between arrays.  

**Figure 2** (PDF file) shows a bar chart of the sum of distances to other arrays *Sa*, the outlier detection criterion from the previous figure. The bars are shown in the original order of the arrays. Based on the distribution of the values across all arrays, a threshold of 14.6 was determined, which is indicated by the vertical line. 5 arrays exceeded the threshold and were considered outliers.

- Figure 3: Principal Component Analysis.  

|  |  |  |  |  |  |  |  |  |  |
| --- | --- | --- | --- | --- | --- | --- | --- | --- | --- |
| xml version="1.0" encoding="UTF-8"? | |  |  | | --- | --- | | array |  | | sampleNames |  | | sample |  | | ScanDate |  | |

  
**Figure 3** (PDF file) shows a scatterplot of the arrays along the first two principal components. You can use this plot to explore if the arrays cluster, and whether this is according to an intended experimental factor (you can indicate such a factor by color using the 'intgroup' argument), or according to unintended causes such as batch effects. Move the mouse over the points to see the sample names.  
Principal component analysis is a dimension reduction and visualisation technique that is here used to project the multivariate data vector of each array into a two-dimensional plot, such that the spatial arrangement of the points in the plot reflects the overall data (dis)similarity between the arrays.

---

## Section 2: Array intensity distributions

- Figure 4: Boxplots.  

**Figure 4** (PDF file) shows boxplots representing summaries of the signal intensity distributions of the arrays. Each box corresponds to one array. Typically, one expects the boxes to have similar positions and widths. If the distribution of an array is very different from the others, this may indicate an experimental problem. Outlier detection was performed by computing the Kolmogorov-Smirnov statistic *Ka* between each array's distribution and the distribution of the pooled data.

+ Figure 5: Outlier detection for Boxplots.  

**Figure 5** (PDF file) shows a bar chart of the Kolmogorov-Smirnov statistic *Ka*, the outlier detection criterion from the previous figure. The bars are shown in the original order of the arrays. Based on the distribution of the values across all arrays, a threshold of 0.142 was determined, which is indicated by the vertical line. 3 arrays exceeded the threshold and were considered outliers.

- Figure 6: Density plots.  

|  |  |  |  |  |  |  |  |  |  |
| --- | --- | --- | --- | --- | --- | --- | --- | --- | --- |
| xml version="1.0" encoding="UTF-8"? | |  |  | | --- | --- | | array |  | | sampleNames |  | | sample |  | | ScanDate |  | |

  
**Figure 6** (PDF file) shows density estimates (smoothed histograms) of the data. Typically, the distributions of the arrays should have similar shapes and ranges. Arrays whose distributions are very different from the others should be considered for possible problems. Various features of the distributions can be indicative of quality related phenomena. For instance, high levels of background will shift an array's distribution to the right. Lack of signal diminishes its right right tail. A bulge at the upper end of the intensity range often indicates signal saturation.

---

## Section 3: Variance mean dependence

- Figure 7: Standard deviation versus rank of the mean.  

**Figure 7** (PDF file) shows a density plot of the standard deviation of the intensities across arrays on the *y*-axis versus the rank of their mean on the *x*-axis. The red dots, connected by lines, show the running median of the standard deviation. After normalisation and transformation to a logarithm(-like) scale, one typically expects the red line to be approximately horizontal, that is, show no substantial trend. In some cases, a hump on the right hand of the x-axis can be observed and is symptomatic of a saturation of the intensities.

---

## Section 4: Affymetrix specific plots

- Figure 8: Relative Log Expression (RLE).  

**Figure 8** (PDF file) shows the *Relative Log Expression (RLE)* plot. Arrays whose boxes are centered away from 0 and/or are more spread out are potentially problematic. Outlier detection was performed by computing the Kolmogorov-Smirnov statistic *Ra* between each array's RLE values and the pooled, overall distribution of RLE values.

+ Figure 9: Outlier detection for Relative Log Expression (RLE).  

**Figure 9** (PDF file) shows a bar chart of the Kolmogorov-Smirnov statistic *Ra* of the RLE values, the outlier detection criterion from the previous figure. The bars are shown in the original order of the arrays. Based on the distribution of the values across all arrays, a threshold of 0.108 was determined, which is indicated by the vertical line. One array exceeded the threshold and was considered an outlier.

- Figure 10: Normalized Unscaled Standard Error (NUSE).  

**Figure 10** (PDF file) shows the *Normalized Unscaled Standard Error (NUSE)* plot. For each array, the boxes should be centered around 1. An array were the values are elevated relative to the other arrays is typically of lower quality. Outlier detection was performed by computing the 75% quantile *Na* of each array's NUSE values and looking for arrays with large *Na*.

+ Figure 11: Outlier detection for Normalized Unscaled Standard Error (NUSE).  

**Figure 11** (PDF file) shows a bar chart of the *Na*, the outlier detection criterion from the previous figure. The bars are shown in the original order of the arrays. Based on the distribution of the values across all arrays, a threshold of 1.04 was determined, which is indicated by the vertical line. 3 arrays exceeded the threshold and were considered outliers.

- Figure 12: RNA digestion plot.  

|  |  |  |  |  |  |  |  |  |  |
| --- | --- | --- | --- | --- | --- | --- | --- | --- | --- |
| xml version="1.0" encoding="UTF-8"? | |  |  | | --- | --- | | array |  | | sampleNames |  | | sample |  | | ScanDate |  | |

  
**Figure 12** (PDF file) shows the *RNA digestion* plot. The shown values are computed from the preprocessed data (after background correction and quantile normalisation). Each array is represented by a single line; move the mouse over the lines to see their corresponding sample names. The plot can be used to identify array(s) that have a slope very different from the others. This could indicate that the RNA used for that array has been handled differently from what was done for the other arrays.

- Figure 13: Perfect matches and mismatches.  

Figure  shows the density distributions of the log2 intensities grouped by the matching type of the probes. The blue line shows a density estimate (smoothed histogram) from intensities of perfect match probes (PM), the grey line, one from the mismatch probes (MM). We expect that MM probes have poorer hybridization than PM probes, and thus that the PM curve be to the right of the MM curve.

---

## Section 5: Individual array quality

- Figure 14: MA plots.  

**Figure 14** (PDF file) shows MA plots. M and A are defined as:  
M = log2(I1) - log2(I2)  
A = 1/2 (log2(I1)+log2(I2)),  
where I1 is the intensity of the array studied, and I2 is the intensity of a "pseudo"-array that consists of the median across arrays. Typically, we expect the mass of the distribution in an MA plot to be concentrated along the M = 0 axis, and there should be no trend in M as a function of A. If there is a trend in the lower range of A, this often indicates that the arrays have different background intensities; this may be addressed by background correction. A trend in the upper range of A can indicate saturation of the measurements; in mild cases, this may be addressed by non-linear normalisation (e.g. quantile normalisation).  
Outlier detection was performed by computing Hoeffding's statistic *Da* on the joint distribution of A and M for each array. Shown are first the 4 arrays with the highest values of *Da*, then the 4 arrays with the lowest values. The value of *Da* is shown in the panel headings. One array had *Da*>0.15 and was marked as outlier. For more information on Hoeffing's *D*-statistic, please see the manual page of the function hoeffd in the Hmisc package.

+ Figure 15: Outlier detection for MA plots.  

**Figure 15** (PDF file) shows a bar chart of the *Da*, the outlier detection criterion from the previous figure. The bars are shown in the original order of the arrays. A threshold of 0.15 was used, which is indicated by the vertical line. One array exceeded the threshold and was considered an outlier.

- Figure 16: Spatial distribution of M.  

**Figure 16** (PDF file) shows false color representations of the arrays' spatial distributions of feature intensities (M). Normally, when the features are distributed randomly on the arrays, one expects to see a uniform distribution; control features with particularly high or low intensities may stand out. The color scale is proportional to the ranks of the probe intensities. Note that the rank scale has the potential to amplify patterns that are small in amplitude but systematic within an array. It is possible to switch off the rank scaling by modifying the argument scale in the call of the aqm.spatial function.  
Outlier detection was performed by computing *Fa* , the sum of the absolutes value of low frequency Fourier coefficients, as a measure of large scale spatial structures.Shown are first the 4 arrays with the highest values of *Fa*, then the 4 arrays with the lowest values. The value of *Fa* is shown in the panel headings.

+ Figure 17: Outlier detection for Spatial distribution of M.  

**Figure 17** (PDF file) shows a bar chart of the *Fa*, the outlier detection criterion from the previous figure. The bars are shown in the original order of the arrays. Based on the distribution of the values across all arrays, a threshold of 0.18 was determined, which is indicated by the vertical line. None of the arrays exceeded the threshold and was considered an outlier.

---

This report has been created with arrayQualityMetrics 3.16.0 under R version 3.0.1 (2013-05-16).

---

  
